# Effectiveness of a Culturally Tailored HIV and Sexually Transmitted Infection Prevention Intervention for Black Women in Community Supervision Programs: A Randomized Clinical Trial

**DOI:** 10.1001/jamanetworkopen.2021.5226

**Published:** 2021-04-09

**Authors:** Louisa Gilbert, Dawn Goddard-Eckrich, Mingway Chang, Timothy Hunt, Elwin Wu, Karen Johnson, Stanley Richards, Sharun Goodwin, Richard Tibbetts, Lisa R. Metsch, Nabila El-Bassel

**Affiliations:** 1 Social Intervention Group, Columbia University School of Social Work, New York, New York; 2 University of Alabama School of Social Work, Tuscaloosa; 3 Fortune Society, Long Island City, New York; 4 New York City Department of Probation, New York, New York; 5 Sociomedical Sciences, Mailman School of Public Health, Columbia University, New York, New York

## Abstract

**Importance:**

Concentrated epidemics of HIV and sexually transmitted infections (STIs) have persisted among Black women in community supervision programs (CSPs) in the United States. Accumulating research has highlighted the effectiveness of culturally tailored HIV/STI interventions for Black women; however, there is a dearth of such interventions for the large number of Black women in CSPs.

**Objective:**

To determine the effectiveness of a 5-session culturally tailored group-based intervention (Empowering African-American Women on the Road to Health [E-WORTH]) with individualized computerized modules and streamlined HIV testing in reducing STIs and condomless sex vs a 1-session streamlined HIV testing control condition.

**Design, Setting, and Participants:**

This randomized clinical trial was conducted from November 18, 2015, (first recruitment) to August 20, 2019 (last 12-month follow-up). Black women mandated to probation, parole, or alternative-to-incarceration programs in New York City who had a history of drug use were recruited and randomized to receive either E-WORTH or a streamlined HIV testing control condition. Both conditions were delivered by Black female staff at a large CSP. The analysis took an intention-to-treat approach.

**Intervention:**

E-WORTH included a 1-hour individual HIV testing and orientation session and 4 weekly 90-minute group sessions. The control condition included one 30-minute session of HIV testing and information.

**Main Outcomes and Measures:**

Primary outcomes were incidence of any STI (biologically assayed chlamydia, gonorrhea, and *Trichomonas vaginalis*) at the 12-month assessment and the number of condomless acts of vaginal or anal intercourse in the past 90 days during the 12-month period.

**Results:**

A total of 352 participants who identified as Black or African American were enrolled, including 79 (22.5%) who also identified as Latinx. The mean (SD) age was 32.4 (11.0) years. A total of 172 participants (48.9%) were assigned to the E-WORTH condition, and 180 (51.1%) were assigned to the control condition. Compared with control participants, participants assigned to the E-WORTH condition had 54% lower odds of testing positive for any STI at the 12-month follow-up (odds ratio, 0.46; 95% CI, 0.25-0.88; *P* = .01) and reported 38% fewer acts of condomless vaginal or anal intercourse during the 12-month period (incidence rate ratio, 0.62; 95% CI, 0.39-0.97; *P* = .04).

**Conclusions and Relevance:**

The magnitudes of effects found across biological and behavioral outcomes in this randomized clinical trial indicate the feasibility and effectiveness of implementing E-WORTH in real-world CSPs. The findings lend further evidence to the promise of culturally tailored HIV/STI interventions for Black women.

**Trial Registration:**

ClinicalTrials.gov Identifier: NCT02391233

## Key Points

**Question:**

Is E-WORTH, a culturally tailored HIV and sexually transmitted infection (STI) prevention intervention, effective in reducing STIs and condomless sex among Black women in community supervision programs (CSPs)?

**Findings:**

In this randomized clinical trial of 352 women, participants assigned to the E-WORTH condition, compared with those in an HIV testing–only control condition, had 54% lower odds of testing positive for any STI at the 12-month follow-up and reported 38% fewer acts of condomless vaginal or anal intercourse during the 12-month period.

**Meaning:**

The findings suggest that implementing a culturally tailored HIV/STI intervention designed for Black women in CSPs is feasible and effective and holds promise for reducing the disproportionate burden of HIV/STIs in this population.

## Teaser

This randomized clinical trial evaluates the effectiveness of a 5-session culturally tailored group-based intervention with individualized computerized modules and streamlined HIV testing in reducing STIs and condomless sex among Black women in community supervision programs.

## Introduction

Black justice-involved women in the United States continue to be heavily impacted by concentrated epidemics of HIV and sexually transmitted infections (STIs). Compared with non-Hispanic White women in the United States, Black women had 23.1 times the rate of new HIV infections in 2018.^1^ These disproportionately high HIV infection rates among Black women are due to myriad factors rooted in structural racism. These factors include discrimination from health care and social service workers, racial inequities in access to HIV treatment, residential segregation, segregated social and sexual networks, racialized drug laws, and mass incarceration. Racialized drug laws (eg, crack sentencing laws that resulted in sentences 100 times longer than cocaine sentences) and racialized policing practices^2,3,4^ have led to a vast overrepresentation of Black individuals in community supervision programs (CSPs), the largest segment of the criminal justice system^5^ that includes probation, parole, and alternative-to-incarceration (ATI) programs. In 2016, 1 of every 23 Black adults in the United States was mandated to CSPs,^6^ which is 3.5 times higher than the rate among non-Hispanic White adults.^6^ Studies have found extremely high rates of HIV (as high as 17%) and STIs (as high as 25%) among samples of all or mostly Black women in CSPs.^7,8,9,10^ Moreover, an estimated 45% of women in CSPs have a substance use disorder (SUD),^5^ a well-established risk factor for HIV/STIs.^11^ These statistics indicate an urgent public health need for effective interventions to reverse the concentrated HIV/STI and SUD epidemics among Black women in CSPs.

Accumulating research indicates the efficacy of culturally tailored interventions in reducing HIV/STIs among individuals from underrepresented minority groups^2,3,4,5,6,7,8,9,10,11,12,13,14^ and Black women in particular.^15,16,17,18^ However, there remains a dearth of HIV interventions designed for Black women in CSPs. A systematic review of 42 HIV interventions in criminal justice settings found that only 7 were delivered in CSPs and only 1 of the 7 was for women,^19^ but it was not culturally tailored for Black women.

To address this gap in culturally tailored interventions, we conducted a randomized clinical trial to test the effectiveness of implementing Empowering African-American Women on the Road to Health (E-WORTH), a group-based HIV/STI intervention with computerized self-paced modules designed for Black women with a drug history who are currently in CSPs. E-WORTH is a new cultural adaptation of WORTH, a US Centers for Disease Control and Prevention (CDC) best practice HIV/STI intervention for women who use drugs^20,21,22^ that we developed for this study using a community-based participatory research methods.^23^ E-WORTH is woven with Afrocentric themes of historical trauma and resiliency stemming from slavery to Jim Crow to the mass incarceration of Black individuals.^2,3,4^ E-WORTH is designed to be delivered in low-resource CSPs. The streamlined HIV testing intervention, which served as the control condition, is recommended by the CDC as an evidence-based best practice HIV testing intervention^24,25,26^ and meets the criteria as a feasible cost-effective standard of care for a control condition. We hypothesized that E-WORTH would be more effective than the streamlined HIV testing condition in reducing any biologically confirmed STI (ie, chlamydia, gonorrhea, and *Trichomonas vaginalis*) at the 12-month assessment and in reducing the number of condomless sex acts during the 12-month follow-up period.

## Methods

### Setting

We conducted the study from November 18, 2015, through August 20, 2019, among 352 Black women who were recruited from CSPs in New York City. The intervention sessions for both study groups were delivered at 2 sites by Black female staff in a large community-based organization, which serves more than 8000 individuals in CSPs annually at 2 New York City sites. The study was approved by the Columbia University institutional review board. All participants provided their written informed consent. This study followed the Consolidated Standards of Reporting Trials (CONSORT) reporting guideline, and the trial protocol appears in Supplement 1.

### Study Design

We used a 2-group hybrid type 1 randomized clinical trial to test the effectiveness of E-WORTH vs a streamlined HIV testing–only control condition. Power analyses showed that a sample size of 372 women (186 per study group) was necessary to achieve 80% power to detect a clinically meaningful 50% reduction in STI incidence, taking into account 20% attrition at 12-month follow-up. The study flowchart is presented in the Figure. Although the actual sample size of 352 women was lower than the estimate projected from the power analyses, the higher-than-expected retention rate and higher-than-expected baseline STI rate yielded sufficient power to detect a clinically meaningful reduction in STI difference

**Figure.**
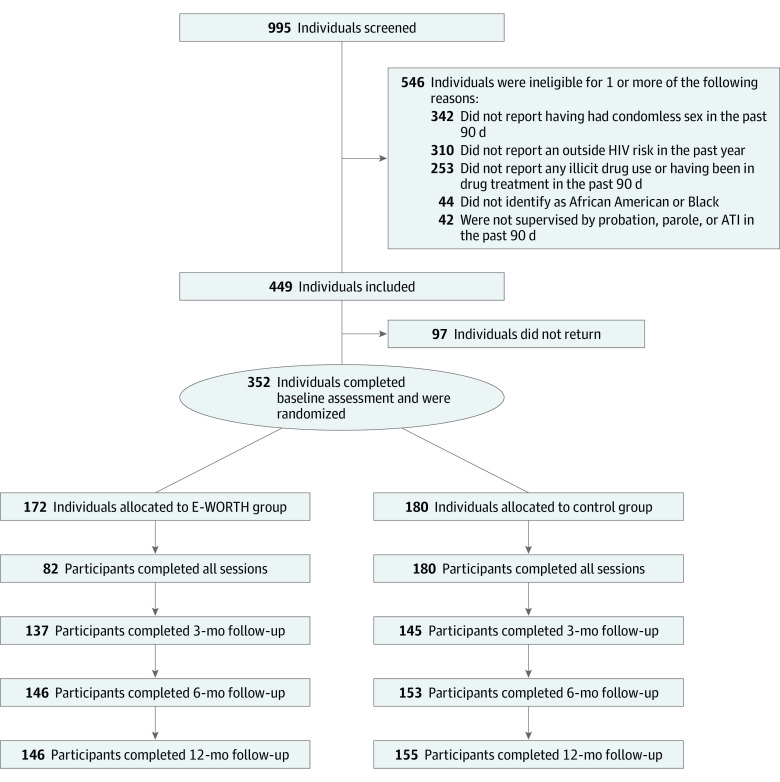
Study Flowchart ATI indicates alternative-to-incarceration.

### Eligibility and Participant Flow

We recruited participants on-site at CSPs and through word-of-mouth referrals. We enrolled most participants (250 [71.0%]) from New York City probation sites, representing nearly 1 in 8 Black women (n = 1808) who were on probation in New York City in 2015.^27^ Women were screened and eligible to participate if they (1) were 18 years or older; (2) identified as African American and/or Black; (3) had been charged with a misdemeanor and/or felony and served a CSP sentence in the past 90 days; (4) reported any binge drinking (≥4 drinks at a time), illicit drug use, and/or having been in substance use treatment in the past 90 days; (5) reported any condomless vaginal or anal sex in the past 90 days; and (6) reported other HIV/STI risks in the past year (eg, sex with multiple partners, syringe sharing) and/or being HIV positive. Exclusion criteria included (1) not being fluent in English and/or (2) being unable to provide informed consent.

### Enrollment, Randomization, and Masking

Consistent with an intention-to-treat approach, participants were considered enrolled only after randomization. Allocation sequences for assembling cohorts were generated using an Excel 2013 version 15.0 (Microsoft Corp) spreadsheet managed by the study’s project director who was masked to assignment until the time of randomization. The randomization scheme was concealed from participants, study staff, and investigators until each cohort was assembled. Periods of randomization were followed by periods of time block assignments to recruit a sufficient number of participants for E-WORTH groups (ie, ≥3 individuals). To eliminate bias during periods of time block assignments, we adhered to fixed randomization and assignment windows governed by the number of days it took to enroll enough participants for an E-WORTH group. We balanced intervention assignments first by condition and second by site. There were no differences between conditions on background characteristics or outcomes at baseline, except for baseline STI rates which were higher in the E-WORTH condition. Given that STI results were not available at the time of randomization and there were no differences in any other baseline outcomes, we believe this difference was spurious. We included baseline STI status as a covariate in all outcome models to adjust for this difference.

### Intervention Conditions

#### E-WORTH Condition

Consistent with empowerment theory^28^ and social cognitive theory,^29^ E-WORTH provides participants with opportunities to develop risk reduction self-efficacy and skills. E-WORTH raises awareness of structural racism rooted in slavery and historical responses of resilience among Black women. E-WORTH optimizes group and individual modalities in a hybrid intervention design. The first session includes an individual orientation to E-WORTH with streamlined HIV testing (described in the control condition section). The 4 weekly 90-minute group sessions feature an in-room Black female staff who facilitates group opening and closing activities and include individualized computerized interactive activities with Black women characters. Core components include raising awareness about HIV/STI risks, condom use technical skills, sexual negotiation skills, risk reduction goal setting, increasing social support and linkage to services, intimate partner violence (IPV) screening, safety planning, and referral to IPV services.^23^

#### Streamlined HIV Testing Control Condition

The streamlined HIV testing intervention was tested in the National Institute on Drug Abuse Clinical Trial Network 0032 HIV Testing study and found to be effective and cost-effective in increasing HIV testing^30,31^ in drug treatment programs. The single 30-minute individual HIV testing session included: (1) watching a 5-minute HIV testing information video; (2) reviewing the OraSure testing pamphlet on transmission risks and strategies for reducing risks; (3) taking the rapid OraQuick HIV test; and (4) receiving the test results and a service manual. Free condoms were regularly distributed for both conditions at both CSP sites.

### Facilitators

Black women who were employed as counselors or case managers at the 2 sites were trained to deliver both E-WORTH and streamlined HIV testing control condition to avoid inextricable facilitator effects. Facilitators completed a 4-day training on both conditions and received biweekly supervision from the project director.

### Data Collection and Measurements

Biological assays for HIV and STIs were performed at baseline and at the 12-month follow-up. We also collected behavioral data at baseline and at 3-, 6-, and 12-month follow-ups through audio computer-assisted self-interviews (ACASI). Participants received a maximum of $240 for all interviews. Transportation was covered for intervention sessions. We also collected sociodemographic characteristics on age, race/ethnicity, marital status, education, employment status, homelessness, food insecurity, and history of incarceration.

### Biological Outcomes

#### Primary Biological STI Outcome and HIV Testing

Participants provided vaginal swab specimens that were tested for chlamydia and gonorrhea using a DNA assay (BD ProbeTec ET Amplified DNA Assay; Becton, Dickinson and Co) and *T. vaginalis *using a real-time polymerase chain reaction assay. Participants with reactive HIV/STI test results were linked or referred to treatment. Participants who were treated for STIs by their own medical practitioners were asked to provide proof of treatment by a prescription bottle or note. Of the 109 participants who tested positive for an STI at baseline, 87 (79.8%) had proof of treatment (38 of 47 control participants [80.9%] and 49 of 62 E-WORTH participants [79.0%]). We included STI outcomes for the total sample, consistent with intention-to-treat approach, but also conducted a sensitivity analysis on new STI cases by excluding 22 participants who lacked proof of treatment. We collected saliva specimens using rapid OraQuick ADVANCE Rapid HIV-1/2 Antibody Test.

### Behavioral Outcomes

The primary behavioral outcome was the total number of acts of condomless vaginal and/or anal intercourse across all partners in the prior 90 days. Secondary behavioral outcomes included the number of condomless vaginal or anal sex acts in the prior 90 days with primary partners, the proportion of protected total vaginal or anal sex acts, and having had vaginal or anal sex with more than 1 partner in the past 90 days.

### Statistical Analysis

Descriptive statistics were used to describe baseline background characteristics for the total sample and by study condition and baseline outcomes among the total sample and by study condition. We assessed differences between the 2 study groups for these variables with 2-tailed *t* tests or χ^2^ tests. To examine the effects of E-WORTH, we used an intention-to-treat approach. To test the difference in the new incidence of STIs at the 12-month follow-up between the 2 study groups, we used logistic regression models that included age, high school graduate (yes or no), employed (yes or no), marital status (married or common law vs single), and confirmed STI at the baseline as covariate adjustments. For behavioral risk outcomes, we used mixed-effects generalized linear models and included a random effect for repeated measures. To estimate the effects over the entire follow-up period, we used the mixed-effects model, including intervention condition, measures of the outcome variable reported at baseline, and the same covariates listed previously. We then obtained the effects from the coefficients associated with the intervention condition. To estimate the effects at each follow-up time point, we included intervention condition, follow-up time, and interaction between intervention condition and follow-up time, baseline measures of the outcome, and covariate adjustment in the mixed-effects models. We then obtained the effects at each follow-up by linear combinations of the coefficient associated with the intervention condition plus the coefficient for the interaction between intervention condition and respective follow-up point. Hypothesis testing for intervention effects on the number of condomless sex acts was based on incident rate ratios (IRRs) from mixed-effects negative binomial regression models. The differences indicated by regression coefficients (b) from mixed-effects linear regression were used for proportion of protected acts of intercourse. Odds ratios (ORs) from mixed-effects logistic regression models were obtained to test intervention effects for the other behavioral outcomes. Statistical significance was assessed using 95% confidence intervals (CI) and *P* value for each estimate. Statistical analyses were performed using Stata 15 (StataCorp).

There were missing data due to loss at follow-up: 70 participants (19.9%) at 3 months, 53 participants (15.1%) at 6 months, and 51 participants (14.5%) at 12 months. Sensitivity analyses for the potential impact of missing data relied on using multiple imputation procedures (MI).^32^ MI imputes values for missing data by using the information that we observed or that were available from a participant’s prior assessments (eg, baseline) to calculate predicted values for the unobserved. Our MI procedures involved 60 imputed data sets using the same mixed-effects generalized linear models described previously.

## Results

### Sample Characteristics

The mean (SD) age was 32.4 (11.0) years (Table 1). All participants identified as Black or African American during the screening. More than one-fifth (79 [22.5%]) also identified Latinx. More than half (196 [55.8%]) completed high school or a GED, and only 61 (17.4%) were married. Nearly two-thirds (230 [65.5%]) identified as heterosexual, and 109 (31.1%) identified as bisexual. Less than one-third (103 [29.3%]) were employed. Nearly two-thirds (222 [63.4%]) reported food insecurity, and 69 (19.7%) had been homeless in the past 90 days. Most participants were on probation (250 [71.2%]), 63 (17.9%) were in an ATI program. and 59 (16.8%) were on parole. Of the total sample, 207 (58.8%) reported using illicit drugs, and 151 (42.9%) reported binge drinking in the past 30 days, but only 81 (23.1%) were in substance use treatment in the past 90 days. A total of 172 participants (48.9%) were assigned to the E-WORTH condition, and 180 (51.1%) were assigned to the control condition. No significant differences were found between conditions on these variables, except for baseline STI rates, which were higher in the E-WORTH condition.

**Table 1.** Descriptive Characteristics of the Sample by Study Group at the Baseline Assessment

Characteristic	No. (%)		
	Total (N = 352)^a^	Control (n = 180)^a^	E-WORTH (n = 172)
Age, mean (SD), y	32.4 (11.0)	32.0 (10.8)	32.9 (11.3)
Latinx ethnicity	79 (22.5)	43 (24.0)	36 (21.0)
High school graduate or GED	196 (55.8)	99 (55.3)	97 (56.4)
Marital status			
Married, including common-law marriage	61 (17.4)	25 (14.0)	36 (20.9)
Single, including never married, widowed, separated, and divorced	290 (82.6)	154 (86.0)	136 (79.1)
Sexual orientation			
Heterosexual	230 (65.5)	120 (67.0)	110 (64.0)
Bisexual	109 (31.1)	52 (29.1)	57 (33.1)
Other	12 (3.4)	7 (3.9)	5 (2.9)
Currently employed full or part-time	103 (29.3)	56 (31.3)	47 (27.3)
In past 90 d			
Homeless	69 (19.7)	29 (16.2)	40 (23.3)
Food insecure	222 (63.4)	109 (61.2)	113 (65.7)
In jail or prison	80 (22.8)	37 (20.7)	43 (25)
On parole	59 (16.8)	29 (16.2)	30 (17.4)
In an alternative-to-incarceration program	63 (18.0)	30 (16.8)	33 (19.2)
On probation	250 (71.2)	126 (70.4)	124 (72.1)
Received alcohol or drug treatment	81 (23.1)	36 (20.1)	45 (26.2)
Binge drinking			
Ever	244 (69.5)	127 (71.0)	117 (68.0)
Past 30 d	151 (43.0)	77 (43.0)	74 (43.0)
Used any illicit drug			
Ever	293 (83.5)	144 (80.5)	149 (86.6)
Past 30 d	207 (59.0)	105 (58.7)	102 (59.3)
Used crack or cocaine			
Ever	96 (37.4)	44 (24.6)	52 (30.2)
Past 30 d	35 (10.0)	19 (10.6)	16 (9.3)
Used heroin			
Ever	32 (9.1)	12 (6.7)	20 (11.6)
Past 30 d	12 (3.4)	5 (2.8)	7 (4.1)
Injected drugs			
Ever	17 (4.8)	5 (2.8)	12 (7.0)
Past 90 d	6 (1.7)	3 (1.7)	3 (1.7)

Abbreviations: E-WORTH, Empowering African-American Women on the Road to Health; GED, general educational development.

^a^ One missing case.

Table 2 presents descriptive statistics on HIV status and outcomes at each assessment by intervention assignment. At baseline, 15 participants (4.3%) had reactive HIV tests, and 1 new HIV infection was detected at the 12-month follow-up. There were 62 E-WORTH participants (36.5%) and 47 control participants (26.3%) who tested positive for any STI at baseline. At the 12-month follow-up, 20 E-WORTH participants (15.2%) and 37 control participants (26.1%) tested positive for any STI.

**Table 2.** Descriptive Statistics of the Outcome Measures at the Baseline and Follow-up Assessments by Study Condition

Outcome	Participants, No. (%)			
	Baseline	3-mo	6-mo	12-mo
HIV^a^				
Control	6 (3.4)	NA	NA	0
E-WORTH	9 (5.2)	NA	NA	1 (0.8)
Chlamydia^a^				
Control	9 (5.1)	NA	NA	8 (5.4)
E-WORTH	9 (5.3)	NA	NA	5 (3.6)
Gonorrhea^a^				
Control	4 (2.3)	NA	NA	2 (1.4)
E-WORTH	2 (1.2)	NA	NA	4 (2.9)
*Trichomonas vaginalis*				
Control	38 (21.2)^b^	NA	NA	30 (21.1)^b^
E-WORTH	58 (34.1)^b^	NA	NA	13 (9.9)^b^
Any STI (chlamydia, gonorrhea, or *T. vaginalis*)				
Control	47 (26.3)^c^	NA	NA	37 (26.1)^c^
E-WORTH	62 (36.5)^c^	NA	NA	20 (15.2)^c^
In past 90 d				
Unprotected vaginal or anal intercourse with all partners, mean (SD), No.				
Control	23.8 (30.6)	17.9 (28.1)	18.4 (29.3)	20.0 (30.3)
E-WORTH	19.4 (27.7)	14.7 (27.4)	16.1 (29.3)	14.3 (28.3)
Proportion of protected vaginal or anal intercourse with all partners, mean (SD)				
Control	0.31 (0.39)	0.43 (0.45)^c^	0.44 (0.48)^c^	0.43 (0.47)^c^
E-WORTH	0.38 (0.41)	0.56 (0.46)^c^	0.55 (0.46)^c^	0.56 (0.46)^c^
Always protected vaginal and/or anal intercourse with all partners				
Control	32 (17.9)	48 (33.1)^c^	59 (38.6)	58 (37.4)^c^
E-WORTH	36 (20.9)	63 (46.7)^c^	69 (47.3)	71 (48.6)^c^
Unprotected vaginal and/or anal intercourse with main partner, mean (SD), No.				
Control	21.1 (30.0)	16.2 (26.8)	17.5 (28.6)	19.0 (29.9)
E-WORTH	17.4 (27.6)	13.9 (27.3)	15.0 (27.9)	13.2 (27.5)
Proportion of protected vaginal or anal intercourse with main partner, mean (SD)				
Control	0.33 (0.43)	0.42 (0.47)^b^	0.45 (0.49)	0.44 (0.49)^c^
E-WORTH	0.42 (0.45)	0.57 (0.47)^b^	0.55 (0.47)	0.56 (0.48)^c^
Always protected vaginal and/or anal intercourse with main partner				
Control	45 (25.1)	54 (37.2)^*^	64 (41.8)	64 (41.43)
E-WORTH	55 (32.0)	71 (51.8)^*^	72 (49.3)	76 (52.1)
Had ≥1 sex partner				
Control	88 (49.2)	45 (31.0)	42 (27.5)	41 (26.5)
E-WORTH	83 (48.3)	36 (26.3)	35 (24.0)	31 (21.2)
Exchanged sex for money or drugs				
Control	30 (16.8)	12 (8.3)	11 (7.2)	16 (10.3)
E-WORTH	31 (18.0)	15 (11.0)	16 (11.0)	13 (8.9)

Abbreviations: E-WORTH, Empowering African-American Women on the Road to Health; NA, not applicable; STI, sexually transmitted infection.

^a^ The reports of HIV/STI at 12-month follow-up represent the new infection cases.

^b^ *P* < .01 by 2-tailed *t* test or χ^2^ test between 2 groups.

^c^ *P* < .05 by 2-tailed *t* test or χ^2^ test between 2 groups.

Compared with control participants, E-WORTH participants had 54% lower odds for detecting any STI at the 12-month follow-up (OR, 0.46; 95% CI, 0.25-0.88; *P* = .01) (Table 3). These results were similar after excluding the 20 participants who tested positive for an STI at baseline but did not provide proof of treatment (OR, 0.48; 95% CI, 0.23-0.90; *P* = .02). During the 12-month follow-up, E-WORTH participants reported 38% fewer total acts of condomless sex in the prior 90 days (IRR, 0.62; 95% CI, 0.39-0.97; *P* = .04), and 42% fewer condomless sex acts with their main partners (IRR, 0.58; 95% CI, 0.36-0.92; *P* = .02). Compared with control participants. E-WORTH participants reported 11 percentage points higher acts of protected intercourse across all partners (b = 0.11; 95% CI, 0.03-0.19; *P* = .009) and with their main partners (b = 0.11; 95% CI, 0.02-0.19; *P* = .013) and were more likely to always use condoms with all partners (OR, 2.11; 95% CI, 1.12-3.97; *P* = .02) and with their main partners (OR, 1.97; 95% CI, 1.06-3.69; *P* = .03) over the 12-month follow-up. The sensitivity analysis using MI yielded similar parameter estimates and significant patterns with the complete case results reported previously (data not shown).

**Table 3.** Multilevel Models for the Intervention Effect Estimates at Each Follow-up Assessment and Over the 12-Month Follow-up Period With Complete Sample^a^

Outcome	Entire follow-up	*P* value	At each follow-up					
			3-mo	*P* value	6-mo	*P* value	12-mo	*P* value
Any STI (chlamydia, gonorrhea, or *Trichomonas vaginalis*), OR (95% CI)	NA	NA	NA	NA	NA	NA	0.46 (0.25 to 0.85)^b^	.01
Any confirmed new STI, OR (95% CI)^c^	NA	NA	NA	NA	NA	NA	0.48 (0.25 to 0.90)^b^	.02
In past 90 d								
No. of unprotected acts of vaginal or anal intercourse with all partners, IRR (95% CI)	0.62 (0.39 to 0.97)^b^	.04	0.63 (0.38 to 1.06)	.08	0.62 (0.40 to 0.98)^b^	.04	0.61 (0.35 to 1.04)	.07
Proportion of protected vaginal and/or anal intercourse with all partners, b (95% CI)	0.11 (0.03 to 0.19)^d^	.009	0.10 (0.004 to 0.20)^b^	.04	0.11 (0.02 to 0.19)^b^	.01	0.12 (0.02 to 0.22)^b^	.02
Always protected vaginal and/or anal intercourse with all partners, OR (95% CI)	2.11 (1.12 to 3.97)^b^	.02	2.13 (0.98 to 4.61)	.06	2.12 (1.10 to 4.06)^b^	.02	2.09 (0.94 to 4.61)	.07
No. of unprotected acts of vaginal or anal intercourse with main partner, IRR (95% CI)	0.58 (0.36 to 0.92)^b^	.02	0.60 (0.35 to 1.03)	.07	0.58 (0.36 to 0.94)^b^	.03	0.54 (0.31 to 0.96)^b^	.04
Proportion of protected vaginal and/or anal intercourse with main partner, b (95% CI)	0.11 (0.02 to 0.19)^b^	.01	0.11 (0.02 to 0.21)^b^	.02	0.11 (0.02 to 0.19)^b^	.01	0.10 (−0.01 to 0.20)	.06
Always protected vaginal and/or anal intercourse with main partner, OR (95% CI)	1.97 (1.06 to 3.69)^b^	.03	2.05 (0.95 to 4.41)	.07	2.00 (1.05 to 3.80)^b^	.04	1.90 (0.87 to 4.13)	.11
Had ≥1 sex partner, OR (95% CI)	0.73 (0.42 to 1.25)	.25	0.78 (0.40 to 1.55)	.49	0.74 (0.42 to 1.29)	.29	0.66 (0.30 to 1.43)	.29
Exchanged sex for money or drugs, OR (95% CI)	1.56 (0.59 to 4.13)	.37	2.71 (0.84 to 8.73)	.10	1.83 (0.68 to 4.94)	.23	0.84 (0.21 to 3.28)	.80

Abbreviations: IRR, incident rate ratios; OR, odds ratio; STIs, sexually transmitted infections.

^a^ Covariate adjustment: age, high school education, employment status (employed full-time or part-time vs not employed), marital status (married or common-law vs single), any confirmed STI at baseline, and baseline measure of the outcomes.

^b^ *P* < .05.

^c^ Excluding 20 participants who tested positive at baseline but did not provide confirmation of treatment.

^d^ *P* < .01.

## Discussion

To our knowledge, this randomized clinical trial is the first to show positive effects of a culturally tailored intervention on reducing STI incidence and condomless sex among Black women in CSPs. Compared with control participants, E-WORTH participants had 54% lower odds of testing positive for an STI at the 12-month follow-up and reported 38% fewer acts of condomless sex during the 12-month period. To consider these findings in context, the intervention effect on reducing STI incidence found in this study was stronger than the average effect from a meta-analysis of 17 HIV/STI interventions conducted among Black women, which observed a 19% reduced odds of STI incidence among intervention participants relative to comparison participants.^12^ Another meta-analysis of 45 HIV/STI interventions for women from ethnic minority groups found only 5 significantly reduced STIs by an average effect of 41%.^12^ *T. vaginalis *constituted most STIs. The significant decrease in *T. vaginalis* incidence at the 12-month follow-up largely contributed to the significance of the STI outcome. The magnitudes of both biological and behavioral outcomes found in this study are clinically and statistically meaningful, indicating the effectiveness of the culturally tailored E-WORTH intervention.

Despite the relatively low rate of HIV infection, the high baseline rate of STIs (30.9%), a risk factor for HIV transmission,^33,34^ combined with the high rate of sex with multiple partners (48.5%) suggest that this sample of Black women was at high risk of acquiring HIV. The high rates of food insecurity, homelessness, incarceration, and unemployment found in this study are also established risk factors for HIV/STIs rooted in structural racism. E-WORTH’s explicit focus on structural racism along with its novel hybrid group format led by Black female CSP staff and a computerized individualized tool with Black women characters promoted effective cultural tailoring of content. This tool further ensured standard delivery of HIV/STI prevention activities and lowered the bar for staff training, increasing the promise of future uptake in CSPs.

### Limitations

This study had several limitations. Generalizability of findings is limited to Black women in CSPs in New York City, specifically to 1 large CSP at 2 sites. The significant STI difference between groups arms at baseline was an unexpected finding that we believe was spurious given the robust randomization procedure and receipt of STI results after randomization. The large effect sizes observed across our outcomes further lend confidence in the STI outcome results. Although the eligibility criteria included having condomless sex with a male partner, 31% identified as bisexual. However, this study did not measure risk behaviors with same-sex partners. Despite these limitations, this study has several strengths, including high rates of participation, attendance, and retention that were similar to the rates in the original WORTH trial.^20^ The diverse sample recruited from probation, parole, and ATI programs and the delivery of E-WORTH in a real-world CSP further suggests the feasibility of implementing E-WORTH in a range of CSPs.

## Conclusions

This randomized clinical trial demonstrated the promise of implementing a culturally tailored HIV/STI intervention to Black women in CSPs, consistent with numerous studies documenting the effectiveness of culturally tailored HIV/STI interventions.^12,13,14,15,16,17,18^ There remains an urgent public health need to scale up culturally tailored interventions to redress the concentrated HIV/STI epidemics among Black justice-involved women that continue to be driven by structural racism.

## Notes

Supplement 1. **Trial Protocol and Statistical Analysis Plan**

Supplement 2. **Data Sharing Statement**
