# Supplementary material for: Effectiveness of a Culturally Tailored HIV and Sexually Transmitted Infection Prevention Intervention for Black Women in Community Supervision Programs: A Randomized Clinical Trial
Source: JAMA Netw Open. 2021 Apr 9;4(4):e215226. doi: 10.1001/jamanetworkopen.2021.5226 (PMC8035652; doi:10.1001/jamanetworkopen.2021.5226)
Supplement: Supplement 1. — Trial Protocol and Statistical Analysis Plan [file jamanetwopen-e215226-s001.pdf]

Columbia University Human Subjects Study Description Data

Sheet

Protocol: IRB-AAAN8409 (Y1M00) Protocol Status: Approved Effective Date: 05/14/2015
Expiration Date: 05/13/2016
Originating Department: SSW Social Intervention Group (576000X)
Submitting to: Morningside
Title: Multimedia HIV/STI prevention for Black Drug-involved women in community corrections
Sponsor Protocol Version#:
Abbreviated title: Multimedia WORTH with MM-WORTH -Black Drug-involved women in community corrections
IRB of record: Columbia University Morningside
IRB number used by the
IRB of record:
Protocol Begin Date: 07/01/2014
Protocol End Date: 06/30/2019
Previous Protocol Number: IRB-AAAD5608
07/23/2015
Principal Investigator: Louisa Gilbert (571000X)

Study Description

1. Study Purpose and Rationale

1.1 Purpose: The proposed study is a randomized controlled trial (RCT) that will rigorously evaluate the effectiveness and cost-effectiveness of delivering a multimedia evidence-based intervention (E-WORTH which is an acronym for Empowering African-American Women on the Road to Health) and streamlined HIV testing to prevent HIV and other sexually transmitted infections (STIs) with Black/African-American (hereafter referred to as Black) women drug users in probation, parole or alternative to incarceration programs(ATI hereafter) sites in New York City (NYC), compared to streamlined HIV testing alone. Repeated assessments will occur at baseline and 3, 6, and 12-months post intervention. The primary outcome will be to reduce cumulative incidence of biologically confirmed STIs (i.e., Chlamydia, gonorrhea and trichomonas. This study has the following specific aims:

1.2. Primary Aims
1. To test the comparative effectiveness of E-WORTH versus Streamlined HIV Testing on primary outcomes of decreasing biologically confirmed STIs and the number and proportion of unprotected sexual acts at the 12-month follow-up and secondary outcomes of reducing drug use, increasing utilization of drug treatment, linkage to HIV care and ART adherence (for HIV+ participants), decreasing partner violence and recidivism.
2. To test if the effectiveness of E-WORTH on study outcomes is moderated by client characteristics (e.g. client subgroups defined by socio-demographics and psychosocial characteristics)
3. To estimate the costs and comparative cost-effectiveness of E-WORTH versus Streamlined HIV Testing on STI infection rates, drug use and projected number of HIV cases averted at the 12-month follow-up.

4. To examine qualitatively and quantitatively how multi-level theory-driven factors may influence the fidelity of implementation and effectiveness of E-WORTH and Streamlined HIV testing on study outcomes.

1.3 Rationale: Despite extremely high rates of HIV and STIs that have been found among women on probation, parole or ATI programs nationwide, to date, WORTH is the only intervention that has been found to be efficacious in increasing condom use and reducing sexual risks among justice involved women in the U.S. Our recent study of 337 drug-involved women on probation or community supervision in NYC found that 17% of the 221 Black women tested positive for HIV and 30% tested positive for an STI 3. The HIV rate found among Black women on probation indicates a highly concentrated epidemic, comparable to rates in sub-Saharan Africa; however, no HIV prevention or testing services are provided in NYC probation sites. The proposed study will address this critical gap in services for Black women drug users in probation settings by testing the effectiveness and cost-effectiveness of the 5-session multimedia E-WORTH intervention.

2. Study Design and Statistical Procedures

2.1. Overview of Study Design: This RCT will be conducted with 420 drug-involved Black women recruited from probation, parole or designated ATI program sites in NYC. Women will be randomly assigned to either (1) the 5-session E-WORTH intervention condition that includes an individual Streamlined HIV Testing session or (2) a Streamlined HIV Testing session (See Section C.9). Fortune providers will deliver both intervention conditions at the Fortune sites in Long Island City or Castle Garden in Manhattan. In extenuating circumstances (e.g. participants are located too far away from these sites), Fortune providers may also deliver both conditions at a NYC DOP sites approved by the appropriate DOP Assistant Commissioner or at our community research office. Women assigned to either condition will continue to receive all services provided by probation, parole or designated ATI program staff. Women who test positive for HIV in either condition will receive HIV post-test counseling and linkage to treatment using the CDC Counseling, Testing and Referral (CTR) Protocol. In month 14, recruitment of participants will begin. We will enroll an average of 14 women per month and complete enrollment by month 45. Self-reported data on behavioral outcomes will be collected from participants via audio computer-assisted self-interview (ACASI). The primary outcome will be measured via biological assay for infection by Neisseria gonorrhea, Trichomonas, and Chlamydia trachomatis, the most common STIs found among women in NYC.

Repeated measurements will take place at baseline and at 3, 6, and 12 months' post-intervention. For Aim 3, we will collect cost data from a variety of sources every six months from months 14-55. At midpoint during enrollment (Month 23), we will conduct a qualitative study on multi-level factors for Aim 4.

2.2 Statistical Procedures: The analytic approach is built upon the investigative team's prior HIV prevention RCTs, which involves an intention-to-treat approach, handling missing data using multiple imputation, and conducting sensitivity analyses to quantify robustness of findings based on models and their assumptions. Descriptive statistics will characterize the sample and measurement distributions to ensure proper application of multivariate methods. We will use multiple imputation to handle missing data. Attrition analyses will identify variables to be

included in MI models to handle nonignorable nonresponse. Preliminary analyses include bivariate analyses to identify unadjusted associations among variables. Variables significantly associated with outcome variables and attrition, as well as significantly different across study arms, will be included as covariates during hypothesis testing. Generalized Linear Mixed Models (GLMM) and Generalized Estimating Equations (GEE) twill be used to account for the nonindependence in measures arising from: (1) “autocorrelation” due to repeated measures with the same person; and (2) “intraclass correlation [ICC]” arising from group membership. The data analytic approach for testing hypotheses with outcome specific models for primary outcomes is presented in Table 4. Analytic strategies for secondary outcomes will follow a similar approach.

Hypothesis testing will implement an intention-to-treat approach. We will employ Generalized Linear Mixed Models (GLMM) and Generalized Estimating Equations (GEE) to account for the non-independence in measures arising from: (1) "autocorrelation" due to repeated measures with the same person; and (2) "intraclass correlation [ICC]" arising from partners who are reporting on conjoint behaviors. Hypothesis testing for Aim 1 follows the form “Assignment to E-WORTH will be associated with significantly lower|higher outcome variable Y at the 12-month follow-up compared to assignment to Streamlined HIV Testing” where ‘lower’ or ‘higher’ is chosen in the direction of reduced risk. The selection of outcome models build upon g (E [(Y_ijx_ij)])=x'_ij where Y_ij is the outcome of interest for the ith participant at the jth time point. The multilevel nature of the data is accounted for by modeling each predictor variable’s coefficient in the vector as dependent on group membership: where the ith participant is a member of the gth group that determines the group characteristics (i.e., elements of the vector w) used to estimate model fit parameters in vector. The components of will be treatment assignment, baseline attribute for the outcome variable, and those variables selected during preliminary analyses noted earlier. The g () function represents a link function that can model the outcome as a function of measurement unit or distribution.

2.3 Power Analyses: From ICC estimates based on the previous WORTH trial, variance inflation factors were calculated to generate effective sample sizes used in G*Power for Aim 1. For cumulative STI incidence, we assumed a 50% reduction (on a background cumulative incidence of 20.5%) to be clinically meaningful to detect via Poisson regression; for behavioral outcomes, projected means, standard deviations, allow for effect size f2 to be calculated and used in G*Power. Results for primary outcomes (Figure 3) indicate that 80% power is achieved for the rate-limiting outcome (i.e., cumulative STI incidence) with an effective sample size of 372 participants; translating to a sample size of 378 participants after accounting for ICC. We then increased the starting sample size to 420 to safeguard against attrition (modeled as a complete loss of 10% of data as conservative estimate since the efficacy trial of WORTH achieved a 91% retention rate at 12-month follow-up and many of those cases provided data at earlier assessment points, thus were not “fully” missing). For secondary outcomes, power analyses participants/arm indicate 80% power to detect differences for effect sizes f2 = .03 to .04 (for = .1 TO .5) with sample sizes as low as 369.

3. Study Procedures

3.1. The Study Team

This research will be undertaken by the Social Intervention Group (SIG) at the Columbia University School of Social Work. SIG is a multidisciplinary research center with a strong infrastructure and experienced researchers and staff. This study benefits from SIG's extensive

clinical trials experience, an investigative team with longstanding collaborative relationships, and the availability of established and tested protocols from previous randomized controlled trials conducted in community correction sites. The investigative team successfully completed an efficacy trial of Multimedia E-WORTH intervention with drug-involved women under community supervision with the New York City Department of Probation. The investigative team will replicate many of the approaches, procedures, and protocols that achieved successful during the pilot trial, which also did not have any adverse events. The study research team will include a project director, a data manager, a clinical supervisor who is a graduate research assistant and 2 research assistants who will be responsible for recruitment, administering assessments and retention of participants. For this study, we will have a service agreement contract with Fortune Society who will hire 6 providers (or 1 full-time equivalent) to conduct the interventions for both study arms. They will not be involved in obtaining informed consent or collecting any data for the study from participants. Fortune Society has multiple longstanding service contracts with the New York City Department of Probation. We will recruit participants from probation, parole or designated ATI program sites located throughout in New York City. We have successfully collaborated with the New York City Department on three randomized controlled trials since 2009. Potential participants in ATI programs or parole will NOT include individuals who are detained in a residential facility or treatment center as a form of sentencing or alternative to incarceration.

3.2. Overview of Study Participation

This randomized controlled trial will be conducted with 420 drug-involved Black women from probation, parole or designated ATI program sites located throughout communities in NYC heavily affected by HIV and STIs. Eligible women will be randomly assigned to: (1) E-WORTH consisting of an individual evidence-based Streamlined HIV Testing session followed by a 4-session group-based multimedia HIV intervention (E-WORTH) or (2) an individual Streamlined HIV Testing session alone (Streamlined HI Testing), which will serve as the comparison condition. Both conditions will be delivered at two Fortune Society Sites (located in Harlem and in Long Island City). If participants live too far to attend sessions at these sites, Fortune staff may also deliver intervention sessions at our community research site or at a NYC DOP site approved by the appropriate DOP Assistant Commissioner. Repeated assessments will occur at baseline and 3, 6, and 12-months post intervention. For participants residing out of New York City during their follow up period, or are otherwise prevented from traveling to study sites to complete a follow up assessment, we will send a link to follow up assessments directly to the participant's e-mail on a password protected PDF document. We will conduct repeated assessments and qualitative in-depth interviews on multi- level factors that may influence the effectiveness of E-WORTH and Streamlined HIV Testing on study outcomes with Fortune providers delivering the interventions, frontline probation, parole or designated ATI program staff (e.g. probation officers), probation administrators and probation clients. Self-reported data on behavioral outcomes will be collected from participants via audio computer-assisted self- interview (ACASI). Primary outcomes will be measured via biological assay for infection by Neisseria gonorrhea, trichomonas, and Chlamydia trachomatis, the most common STIs found among women in NYC.

We will also conduct qualitative interviews with 20 E-WORTH participants who reported episodes of homelessness and depression when completing assessments at baseline, 3-, 6- and/or 12-month. The goal of these interviews is to better understand the relationship between

homelessness and depression among women involved in the criminal justice system who are also at risk for HIV and other STIs.

We will conduct focus group interviews with EWORTH and Forune society staff to bettwe understand 1) study implementation and barrier; 2) Linkage to care, to **understand barriers and facilitators to better understand care and usages of housing, employment, mental health and sexual health services; 2) Sexual Healtg Literacy to acess community sexual health literacy, what kind of information and where they are getting; 3) Opioid and Community to access opioid use in the community and attitudes towards Medication-Assisted Treatment (MAT) Medication-assisted treatment (MAT), including opioid treatment programs (OTPs), combines behavioral therapy and medications to treat substance use disorders.; 4) Linkage to care barriers to understand barriers and facilitators to better understand care and usages of housing, employment, mental health and sexual health services.**

3.3. Recruitment and Screening of Participants

Recruitment of participants for RCT for Aim 1: Recruitment will follow procedures that have been approved by Columbia University for recruiting participants from probation sites Projects PACT (AAAK0206), WORTH (AAAD5608) and WINGS (AAAK2851). These procedures have been refined over the course of the investigative team's multiple federally-funded studies that have successfully screened over 1000 potential participants and met target enrollment numbers of participants into HIV prevention trials. We will use various strategies for recruitment, including onsite recruitment at probation, parole or designated/selected ATI program sites, field referrals and by word-of-mouth described in detail under F Section.

Screening of participants for RCT: If a woman expresses interest in being screened for the study, the RA will describe the study and answer any questions. All potential participants will be apprised that their involvement in the study is entirely voluntary and that it doesn't fulfill any court mandate. If the woman indicates that she is willing to be screened, the RA will obtain written informed consent to be screened by reading the screening consent form to the participant and asking if she has any questions about the screening interview before obtaining her signature on the form. After obtaining informed consent, the RA will conduct a 10-15-minute screening interview to determine eligibility and willingness to participate. The screening interview will contain eligibility related items, socio-demographics, and other items that will camouflage the eligibility criteria. Interested potential participants will undergo a written informed consent and complete a 10-15-minute screening in a private room, which will consist of questions about sexual behaviors, history of drug use, and demographic information (see screening form). If a private room is not available, or if the participant wishes to be screened at a later time, the participant will be screened via phone. Once the potential participant's eligibility has been established, the RA will schedule her for an appointment to complete the baseline assessment.

The RA will ask participants who screened eligible to complete a contact information sheet that asks for their address, telephone numbers where they can be reached, and email information using a Contact information sheet. More detailed contact information (Locator Form) is obtained when participants come for the baseline assessment. In addition, participants will be asked to provide the number of children they care for directly using the Child Information Form in the event that childcare becomes a barrier to participation. Potential participants will receive a handout that introduces the study, explains compensation, and contains a contact telephone number. To ensure participants are aware of meeting times, we will send letters to remind them

of their baseline assessment, HIV testing and E-WORTH session appointment times. When potential participants are not eligible, we will send a re-screen letter after 90 days to see if they are interested in screening for the study again. At the time of the screening interview, participants will also be asked to sign a HIPAA form.

Participants will be compensated $5 for completing the screening interview. Women who meet eligibility criteria (described below) will be asked to participate in the study, and will be told that their decision whether or not to participate in the study will not affect their status with court, NYC Departments of Probation and Parole, and designated ATI sites, or the criminal justice system. Interested participants will undergo an additional informed consent process, and will be scheduled to complete a pre-intervention (or baseline) assessment at the SIG Community Research Site. During the baseline interview, participants will be asked to provide more detailed contact information using the E-WORTH Locator Interview form. In addition, since the intervention will be delivered electronically, participants will also be asked to fill out an E-WORTH Computer Literacy Questionnaire during the baseline interview to gauge levels of computer literacy. Research Assistants will complete a study check list each time participants are assessed to ensure that all items on the check list are completed.

Recruitment of probation, parole or ATI program staff and Fortune providers for survey (Aim 4)– Prior to the beginning of the study, we will present the purpose and procedures of this study to all probation, parole or ATI program staff at the DOP study sites and to Fortune staff. After our presentation we will ask staff to participate in the survey. Staff, who are interested in participating in the study, will complete written informed consent administered by a research assistant prior to enrolling in the study. During informed consent, potential participants will be apprised that their participation in our study will not affect their position at DOP or Fortune in any way and they may withdraw from the study at any time without questions asked. For the mixed study for Aim 4, we aim to enroll 25 staff probation, parole or ATI program officers and 10 administrators (supervisory Probation, parole or ATI program Officers/Branch Chiefs/Other Managers) and the providers from Fortune Society (1 per site, n=5) delivering E-WORTH and Streamlined HIV Testing condition will be asked to complete ACASI structured survey the month prior to starting the RCT with repeated assessments every 6 months through month 45.

Recruitment of participants and probation, parole or ATI program staff for in-depth interviews (Aim 4) — From all the NYC probation, parole or ATI program sites, the Project Director will randomly select 4 women on probation, parole or ATI program who enrolled in the study (a total of 20 participants) and ask them to complete the in-depth survey. The Project Director will also randomly select 10 probation, parole or ATI program officers and 10 Probation, parole or ATI program Administrators from those who completed the ACASI structured survey and invite them to participate in an in-depth interview. In addition, the Project Director will invite all service providers from Fortune society who delivered both intervention conditions (5 service providers) to participate in an in-depth interview. The project director or an RA will contact all probation, parole or ATI program staff and study participants who have been selected to participate in the in-depth interviews by phone or mail. The project director or an RA will describe the purpose of the in-depth interview and answer any questions that randomly selected staff and study participants may have about the in-depth interviews. If staff and study participants indicate that they are willing to participate in the in-depth interview, the project director or an RA will administer informed consent.

Recruitment of participants for in-depth interviews focusing on homelessness and depression. The Project Director will identify all E-WORTH participants who reported histories of homelessness and depression when completing assessments at baseline, 3-, 6- and 12-month. Recruitment will be conducted under the direction of the Project Director by an RA and a study consultant. Interviews will be held in our offices (at the Social Intervention Group's community research site located at 3280 Broadway, 8th floor) and is expected to last for up to 90 minutes.

3.4. Assessments:
Participant Assessment for Aim 1: When a participant arrives for their pre-intervention assessment, they will undergo a shortened version, 5 minutes, of the screener to confirm eligibility. If a woman no longer meets eligibility criteria, she will be compensated $10 and will no longer participate in the study. Women who meet eligibility criteria will undergo a pre-intervention assessment. This assessment will last approximately 60 minutes, consisting of demographic information, history of criminal justice involvement, drug and alcohol use and dependence, sexual and drug-related HIV behaviors, HIV treatment and care, intimate partner violence, depression and PTSD, service utilization, and social support. RAs will schedule repeated assessments with participants that will take place at baseline and at 3, 6, and 12 months post- intervention. For all assessments, participants will complete the ACASI surveys on computers with headphones at a computer work station designed for privacy so that others cannot see their responses on the computer. Participants will be given the option to complete the baseline survey at SIG's community research office at 3280 Broadway or on site at Probation, parole or designated ATI program or Fortune Society sites.

Process data collected for Aim 4: Providers will collect attendance data for each intervention session using the E-WORTH attendance form and provide information on number and general type of referrals to services using participant ID numbers. Providers will also be asked to complete a session adherence form for each session that assesses the extent to which core elements and activities of the session were covered and the quality of delivery of each activity using a rating scale. At the end of session 5, E-WORTH participants will be asked to complete an anonymous ACASI client evaluation of intervention that assesses their level of satisfaction with the intervention and provide. Streamlined HIV testing alone participants will also be asked by their providers to complete an anonymous self-administered written evaluation of the session. In addition, the computerized multimedia E-WORTH tool will generate data on whether or not participants completed each session activity and the amount of time spent on each activity. Staff survey Assessment for Aim 4: For Aim 4, probation, parole or ATI program officers (n=25), administrators (n=10) and the probation, parole or ATI program providers from Fortune Society (n=10) delivering E-WORTH and Streamlined HIV Testing will be asked to complete an ACASI survey that will take approximately 45 minutes at month 9 with repeated assessments every 6 months through month 45. After completing informed consent, probation, parole or ATI program staff and provider participants will be asked to complete an ACASI survey which they may complete on line using the secure Dat Stat Server described below. This survey will include questions and scales that assess multi-level factors that may influence the fidelity of implementation and effectiveness of both intervention conditions on study outcomes, including: socio-demographics and professional training of staff and providers, their attitudes toward the E-WORTH and streamlined HIV testing condition, provider self- efficacy in delivering interventions, Organizational Readiness to Change, and Organizational Climate.

In-depth interviews of study participants, Fortune providers and staff for Aim 4: To help us open the “black box” of the delivery of E-WORTH and Streamlined HIV Testing intervention in probation, parole or ATI program sites, we will collect qualitative data to identify multi-level factors and processes that enhance or diminish the fidelity of implementation, which would otherwise be unobserved in the quantitative analysis. At the midpoint of trial (Month 23), we will conduct in-depth interviews with 10 Fortune providers who delivered the sessions, 20 probation, parole or ATI program officers, 10 Probation, parole or ATI program administrators, and 10 E-WORTH and 10 HIV testing information participants randomly selected from each site. All interviews will be audio-recorded and transcribed. The project director or GRA for the study will be trained to conduct in-depth interviews with selected Probation, parole or ATI program staff and study participants and Fortune providers who deliver the interventions for both conditions.

The first section of the interview will be administered to all Fortune providers delivering the interventions, probation, parole or ATI program officers and administrative staff to identify multilevel factors or processes that may influence the fidelity of implementation of the interventions, such as: perceptions about the benefits/disadvantages of both interventions for their site, their perceptions of clients’ reactions to both interventions, their perceptions about their probation, parole or designated ATI programs organizational climate, and their attitudes towards criminal justice. The second section will be delivered only to Fortune providers delivering both interventions, which will explore their perceptions about: what core components of E-WORTH are relevant/useful, difficulties they experience in delivering intervention activities, how participants responded to the cultural tailored content of the E-WORTH sessions, group dynamics, barriers to engagement of
participants and their perceptions of what training, technical assistance and supervision is needed to deliver both interventions and any unanticipated issues or incidents that they experienced. In-depth interviews with 20 randomly selected participants will explore their attitudes towards probation, parole or ATI program staff and Fortune providers and any barriers that may have impeded their participation in the intervention.

All qualitative interviews will be audio-recorded and transcribed. We will construct a qualitative dataset that will include identification of each transcript, inclusion of relevant demographic data by provider/probation, parole or ATI program staff and by probation, parole or designated ATI program sites that will aid in data analysis of key themes that emerge. A preliminary set of analytic coding categories (closed codes) will be assembled based on concepts, ideas, themes and patterns that characterize multi-level theory- driven factors or processes described above. This set of codes will be continually updated through a process of contrast and comparison. The initial or open-coded data is then organized under the analytic category list. Qualitative data will be analyzed using the Atlas 5.2 software system. Key themes and patterns related to how multi-level moderators may influence implementation of the intervention from the qualitative findings will be compared and contrasted to the quantitative study results for specific multi-level theory-driven moderators for Aim 3 described below and process measure data on fidelity of implementation. The qualitative data will be used to interpret the outcomes of the study.

Usage data collected from multimedia website: The web program for E-WORTH will collect log in and log out date and time. Any time a resource such as a video or document is accessed, the web program will track the time and date of access, and for videos, the duration and proportion

of resource viewed. Participants will be asked to respond to questions that assess from which type of location they are accessing the web program from and with whom they are viewing the website with.

3.5. Biological Assays

Collection and Testing of Biospecimens for STIs: To complement self-reports and provide a more objective outcome, we use biological assays for gonorrhea, Chlamydia, and trichomonas. These STIs are selected based on their high prevalence and incidence in this population, the availability of accurate tests, and because each can be definitively treated with a single oral dose of antimicrobial medication. We will assess STIs with DNA amplification assays on self-collected vaginal swab specimens (also referred to as STI testing) from the women, which have several advantages including stability for transport and ease of collection. Immediately following the baseline assessment, the clinical research coordinator (CRC) will provide pre-test counseling on STIs to the participant and then ask her to collect a vaginal swab (self-administered by the participant) that will be used to test for gonorrhea, Chlamydia and trichomoniasis. The clinical research coordinator will then provide follow up post-test counseling to notify participants of their results and treatment referrals as needed. Each participant will be asked to obtain one vaginal specimen by inserting a sterile Dacron-tipped swab about 2.5 inches or as far as comfortable into the vagina, rotating it for 15 to 30 seconds, and removing it. The vaginal swabs are placed into separate specimen transport packaging. Refrigerators will be utilized for storing specimens between shipment dates. Specimens will be shipped within 72 hours of collection. All specimens will be shipped and processed by Bioreference Laboratory in New York City using standard handling procedures in specially designed biohazard containers suitable for Dangerous Goods Shipping according to International Air Transport Association (IATA) guidelines. Bioreference has worked on other currently NIH funded studies involving STI testing, including another randomized controlled trial with probationers (Project PACT). We will not conduct biotesting or store biospecimens at DOP sites. Biotesting will be conducted at Fortune locations or at SIG's community research office.

Notification to Participants of STI Results: For delivery of STI results, participants are given a card with a unique study ID number, the name and contact numbers of the CRC and Project Director, and the expected date to contact the CRC for test results. Participants, who test positive for any of the STIs, will be informed privately by the CRC, counseled, and referred for treatment (see treatment protocol below). While all attempts will be made to deliver STI results in person, the CRC will provide STI results over the phone to participants who indicate they are not able to report to the study site in person in an effort to expedite treatment. The research assistant will obtain participant consent to deliver STI results over the phone at baseline. Detected STIs are treated according to CDC guidelines. After notification, the participant will be directed to seek services at her preferred healthcare provider. If participants do not have a health care provider, they will be referred to free STI clinics as well as a healthcare provider that is within a 5-minute walk, has a sliding scale and accepts Medicaid, and has worked with SIG in prior and ongoing HIV/ STI clinical trials.

Biospecimen Testing Procedures for Streamline HIV testing and counseling: Immediately following randomization participants in both conditions will receive HIV testing as part of the Streamline HIV testing intervention and again at the 12-month follow-up assessment. Fortune providers who will be delivering the intervention condition or the CRC at the 12-month follow

up will ask all participants to provide an oral specimen, using the OraQuick test procedures to confirm their HIV serostatus. Sensitivity and specificity for the OraQuick oral fluid assay are 99.3% and 99.8% respectively. Results are available from the rapid test in 15 minutes. If the first OraQuick ADVANCE assay is nonreactive, then no further testing will be conducted. If the rapid test is reactive, then the participant will be informed about the results and a confirmatory OraQuick test will be conducted immediately. Regardless of whether the confirmatory rapid test is reactive or non-reactive, the participant will be informed of the confirmatory results and referred for further confirmatory HIV testing and, if needed, treatment and care. All participants will receive standard post-test HIV counseling from the Provider or the Clinical Research Coordinator, who will be certified to conduct such counseling. Participants who are identified as HIV seropositive will be informed in private and referred to clinical sites for further diagnostic evaluation and care. The collection, results, and referral procedures for HIV and STIs have been used successfully and without adverse events in the investigative team's prior and ongoing federally-funded clinical trials of HIV preventive interventions with at-risk and/or marginalized women. During the informed consent process, we will apprise participants that we will report positive Chlamydia, gonorrhea, or HIV tests to the New York City Department of Health (NYCDOH), and that this is the same procedure followed when people are tested in a private physician's office. We will explain all procedures in the consent form, up front, including potential risks and benefits (especially the benefit of detecting and getting treatment for an STI and HIV, when symptoms are not observable). We have not found this to jeopardize trust in any way. In fact, the openness of the consent form may enhance trust.

Legal HIV/STI Reporting and Tracing Requirements: If a participant tests positive for gonorrhea, Chlamydia, or HIV, the CRC will complete a Confidential Morbidity Report. The "Confidential Morbidity Report," which identifies the STI-positive individual and the specific STI diagnosis, must be received by the NYCDOH within two weeks of the CRC receiving the participant's STI results. Upon the receipt of the Confidential Morbidity Report, NYCDOH will initiate contact tracing as per standard procedures. Upon referral, NYCDOH protocol requires that diagnosed individuals be interviewed by a trained Disease Investigation Specialist and asked about their sexual contacts for contact tracing. NYCDOH will ask infected individuals whether they would like to disclose positive tests to their partner(s) and bring the partner in for treatment, or whether they would prefer that DOH staff contact the partner. Individuals testing positive for STIs, including HIV, however, are not mandated to, nor can they be coerced to, disclose the names of their sexual partners. Both scenarios offer opportunities for the participant to choose whether or not to disclose with or without support.

Protocol for STI Treatment: All participants who test positive for gonorrhea, Chlamydia and trichomoniasis will be referred for treatment with a single dose antibiotic therapy that will be administered by licensed physicians who are participating providers for SIG's current studies, or if the participant prefers, by her primary care provider. All SIG providers accept Medicaid and all other types of insurance. All SIG providers have received human subjects training. If a participant is uninsured and can't afford treatment, SIG will pay for treatment out of in-kind funds. We anticipate that very few participants who test positive for an STI will not have Medicaid or other insurance based on our current studies with similar populations. The participants' STI and HIV serostatus will be kept strictly confidential and known only to the participant, the CRC, and the physician who is treating them. These procedures are being used in our current studies and are in compliance with the most up-to-date New York State and New York City laws and standards. Of critical importance is the confirmation of participant's

treatment. From both ethical and methodological reasons, it is imperative that treatment be completed promptly. Thus, the CRC will work closely with the participant to confirm that STI treatment has been received. After notification, the participant will be treated immediately or scheduled to return to a designated clinical site for treatment by a project clinician. For both ethical and methodological reasons, it is imperative that treatment be completed promptly and confirmation of intervention is obtained. Participants are treated with directly observable state-of-the-art single-dose oral therapy to minimize potential non-adherence to multi-dose medication regimens. We have been successfully using this STI treatment protocol in Project PACT and Project WORTH.

Protocol for linking participants who test positive for HIV to treatment: Receiving a positive test result for HIV may produce psychological stress. The study providers and CRCs will be trained to provide post-test counseling, identify and address any psychological distress associated with receiving a positive HIV result and provide enhanced referrals for HIV care. We will follow current CDC recommended guidelines and evidence-based strategies for ensuring linkage to HIV treatment and care. Specifically, our staff will be trained on the evidence-based ARTAS (Antiretroviral Treatment Access Service) intervention that was shown to effectively link persons newly diagnosed with HIV to primary medical care). Our co-investigator Dr. Lisa Metsch was one of the developers of this interventions and has provided trainings throughout the U.S on this intervention. The Centers for Disease Control and New York City Department of Health currently recommends ARTAS as an active linkage intervention. Participants, who [newly] test HIV positive during the streamlined HIV testing intervention session or at the 12-month follow up assessment, will be offered information about local resources and referred to clinical sites for further diagnostic evaluation and care. Providers or the CRC will help participants identify which HIV care clinical site will work best for them and offer to call the clinic to make an appointment. Providers will also give newly diagnosed participants their contact information and encourage them to call if they have any follow up questions or need additional assistance to access HIV care. Although we offer this HIV testing at no cost to the participant, she may choose not to get tested and still be able to participate in the study (i.e. willingness to take the HIV test is not an eligibility criterion).

3.5. Randomization

Consistent with an intention-to-treat approach for data analyses, participants will only be considered enrolled in the trial when they have been randomly assigned to a study arm. We will implement urn randomization to "force" a balance (without adding bias) between study arms with respect to the number of women per arm so that half of the women will be randomized to E-WORTH and immediately receive the individual Streamlined HIV test session followed by 4 E-WORTH group sessions and half will be randomized to and immediately receive the Streamlined HIV Testing session alone. The randomization program, which has been adapted from our previous RCTs, will also feature a combination of randomization and assignments to balance the number of women per condition per site so that the number of women assigned to each condition will be distributed across probation, parole or designated ATI program and study sites. In addition, participants randomized to the intervention arm will complete the intervention individually if less than 4 participants are enrolled at a given site in 28 calendar days. Additionally, the assignment window during the Treatment as Usual condition assignment phase will open after the first participant is assigned. We will explain the randomization process to participants during informed consent. The randomization and assignment protocol will also

ensure moderators of intervention effects assigned to study arms, thus minimizing the possible introduction of bias that can occur with urn randomization or block permutation techniques.

3.6. Intervention Conditions

Streamlined HIV Testing Alone Condition: The streamlined HIV testing with information Alone condition was tested and found to be effective in two recent large scale multi-site studies that were conducted in the NIDA Clinical Trials Network (CTN 0032 and Project AWARE) and led by our co-investigator Dr. Lisa Metsch. This study will employ the Streamlined HIV Testing protocols used for these trials. Fortune Society staff will deliver a 5-minute HIV testing information session to HIV Testing participants in a private space that will describe the rapid testing procedure, timing for and meaning of test results, explain the window period during which an antibody test might be negative. Providers will briefly describe what HIV infection is and give them the pamphlet from the HIV testing kit, which provides an overview of transmission risks and effective strategies for reducing. This streamlined HIV testing intervention is consistent with New York state law that does not require risk reduction counseling at the time of HIV testing.

Participants are then offered a rapid Oraquick HIV test and escorted to a waiting room for 20 minutes until the test results are ready. For participants who test negative, Fortune providers will conduct another 5-minute explanation of the duration of the window period during which the test might be falsely negative. HIV post-test counseling for participants who test positive for HIV will follow CDC-recommended ARTAS protocol that will include providing psychological support and referral for confirmatory HIV testing and care, identify and address barriers to care, and review plan to reduce risks and discuss disclosure issues. WORTH Intervention Condition: WORTH consists of one Streamlined HIV Testing session described below followed by four 1.5-hour group sessions of 3-8 women that will be delivered by a Fortune Society facilitator provider at the Fortune Society sites. The main multimedia components used in the WORTH: (1) Narrativity: Use of videos with fictional characters who resemble different life stories of Black women affected by HIV/AIDS, drug use and criminal justice. These characters provide inspirational messages, model group support and core risk reduction skills; (2) Skill acquisition using simulated video vignettes that provides instruction and demonstration of core skills (e.g., safer sex negotiation and problem-solving skills, technical condom use skills) using culturally congruent role models followed by simulated video vignettes of role play scenarios where participants are prompted to identify common pitfalls in using skills and to rehearse appropriate responses; (3) Individual interactive exercises and logs that are designed to enhance participant's recall of core knowledge and to provide a confidential space for participants to track their individual progress in reducing risky behaviors and achieving risk reduction goals, current and ideal future social networks, and the types of support for sustaining safer behaviors, and future plans; and (4) Facilitator interactive guide which acts as a road map, prompting the facilitator to move sequentially through each activity without the need to rely on notes or memory. The basic format of each E-WORTH session remains consistent following a sequence of 5 steps: (1) an opening (quote, song, poem) which will provide a brief culturally relevant point of inspiration to engage participants; (2) Check-in to review material from the previous session, (3) a discussion to raise awareness of links between IPV, drug use, criminal justice involvement and HIV risks; (4) a skills-building component relevant to the discussion; and (5) an individual computerized rating of their sexual and drug risks; (6) an individual computerized to set appropriate risk reduction goals and plan of action which they can text to themselves. (7) identification of service needs and appropriate referrals using a computerized service tool. E-WORTH more exclusively tailored to the realities of Black women on probation, parole or ATI programs, including: (1) adding recent HIV/STI statistics for Black women in NYC on probation, parole or an ATI program to raise awareness about the epidemic in their communities; (2) using only Black characters and narratives, incorporating Afro-centric messages that

highlight historic resiliency of African-descended women to enhance protective practices and focus on the unique challenges (e.g., stigma, racism, discrimination) Black drug-involved women on probation, parole or ATI encounter; and 3) upgrade the web-based platform so that it may be used with tablet and mobile phones. See Attached E-WORTH intervention manual with login information to the computerized E-WORTH intervention.

3.7. Compensation

All participants will be reimbursed up to $340 for their time for completing the screening and the assessments. Below is the compensation that participants or potential participants in the randomized controlled trial study will receive for study activities. This includes incentives to cover travel expenses to office sites.

$5 for eligibility screening interview
Two-fare metrocard currently valued at $6
$55 for enrollment (that is $35 for completing the baseline assessment, vaginal swab specimen - also referred to as STI testing, and $20 for orientation and HIV testing)
$20 bio retesting
$50 for getting Bio Results and Bio Treatment in the same day. Transportation and change of contact info is included.
Plus, an extra $5 if the participant arrives on time for the enrollment meeting
$100 ($15.00 for each of the 4 intervention sesessions you complete + an extra $5 for being on-time to each session. You will also receive $6 for transportation.)
$50.00 for completing the 3-month follow-up interview
$60.00 for competing the 6-month follow-up interview
$65.00 for completing the 12-month follow-up interview and STI test and HIV test
$50 CBO referral bonus (10 participants)
$20 participant referral bonus (10 participants)
$-15 childcare
$5 change of contact information
$35 Graduated participants will be invited to have a qualitative interview

They will also receive a light meal at each visit to the office.

In addition, participants and CBO staff are also eligible to earn the following if they refer others to the study:
$5.00 for every person they refer to the study that is later screened. When a CBO staff successfully refers 10 eligible participants who enroll and attend session one, they will receive a bonus of $50. When a participant makes 10 succesful referrals, they will receive a bonus of $20. The person referred must meet the basic criteria of the study. That is, she must be Black, 18 years or older and must be on probation or recently completed probation in the past 90 days.
$10.00 for every person referred to the study who is later enrolled and completes their first follow up meeting.
$15 Childcare incentive will now be offered to women for assessments.
$50 incentive now will be offered to be able to encourage hard to reach participants who were also tested positive for an STI and need to be treated as soon as possible before they are due for 12 month follow up appointment. Hard to reach participant will be provided her STI results and escorted by a Research Asisitant to the most convenient Free STI Clinic. Incentive is included delivering the STI Results, Getting Treated and Confirming the Treatment, Transportation and Changing Contact Information when its all done in the same day.

Lastly, participants are also eligible to receive an additional $5.00 if they change their contact information (i.e. phone number, mailing address, etc…) and report the change to a member of the study.

For the mixed methods study with Fortune Society employees who are assigned to the E-WORTH Project will receive the following compensation for study activities. NYC Department of Probation does not allow their staff to receive any compensation for study activities.

$30 for survey interviews (repeated every six months from the study start to month 56)
$30 for qualitative interview. ($30 + $5 for transportation= $35)

For the in-depth interviews focusing on homelessness and depression, *participants will receive $50 in a gift card and $6.00 round trip metrocard to cover travel expenses.*

3.8. Retention
This study will implement tracking procedures used in our previous research that have proven effective in achieving high retention rates of low income, drug-involved and criminal justice-involved populations. These procedures include: (1) administering a detailed locator package at baseline; (2) assigning cases to RAs to track from baseline to the 3-month follow-up; (3) verifying locator data and updating data at every assessment; (4) maintaining biweekly phone, text, email, or mail contacts with participants during the follow-up period (i.e., birthday cards, holiday cards, appointment notices, thank you cards, check-in calls); (5) implementing returned mail procedures; (6) conducting outreach to unconfirmed cases; and (7) making reminder calls to participants one week prior to their appointment and 24 hours prior to their appointment. In collecting locator information on alternative contacts (i.e., friends, neighbors, family members, and service providers), RAs will apprise participants that they will not disclose their study participation to the contacts, but simply introduce themselves as Columbia University staff and leave a message with the alternative contact for the participant to call them back. RAs will be trained to engage participants in identifying and overcoming barriers to attendance at follow-up sessions. The participants will receive monetary compensation for assessment sessions (see above compensation section or specific details).

3.9. Quality Assurance
All sessions for both conditions will be digitally recorded with 10% randomly selected for QA to rate how closely facilitators adhered to the intervention protocol and the overall clinical quality of delivery each session activity. QA data are reviewed, processed, and addressed/troubleshot during regular Facilitator supervision. If QA data for a Facilitator is found to fall in the insufficient range after attempting to rectify the problem, the Facilitator will not be assigned new couples (but will continue with existing couples to ensure continuity for participants with all recordings reviewed) until the Facilitator has successfully become "recertified" via re-training (e.g., additional readings, mock role-plays). Any detected instance(s) of contamination by facilitators or improperly handled responses to participants bringing up information from the other intervention will trigger immediate corrective action

4.0. Selection and Training of Staff
All study personnel will complete a common training that includes the impetus, goals, organization, protection of human subjects, and rules/regulations. Research Assistants will receive additional hours of structured and standardized training for specialized tasks. Facilitators

from Fortune Society will be trained to deliver both intervention arms. The facilitator training course (one for each condition held at different times) lasts 5 days and modeled on similar trainings conducted during the investigative team's prior and ongoing couples-based HIV prevention RCTs. All study staff will complete all required human subjects trainings designed to ensure the protection of the rights and ethical treatment of human subjects. All personnel involved with or supervising the implementation of biological assays will also complete the blood-borne pathogen/infection control laboratory training. Documentation of sufficient and appropriate human subjects and laboratory training will be achieved by submitting a modification to the IRB protocol with the newly hired and trained staff.

4. Study Drugs or Devices

N/A

5. Study Questionnaires

The following questionnaires are attached to the IRB protocol:

Screening Questionnaire: The eligibility questionnaire will be administered in a face-to-face interview by a trained RA. The screening interview will take approximately 15 minutes. Before completing the baseline, an RA will administer a face-to-face interview with selected eligibility items from the Questionnaire to confirm eligibility of participants.

Baseline/Follow up assessment: This assessment will be used for baseline as well as 3-, 6- and 12-month follow up assessments.
- Immediate post-intervention evaluation questionnaire: This evaluation questionnaire will be administered individually at the end of the final session for E-WORTH participants and at the end of the streamlined HIV testing alone session for participants assigned to this condition. There is an evaluation form specific for both intervention arms.

6. Study Subjects

We plan to enroll 420 women in each of three intervention conditions, for a total of 420 women. In order to obtain the desired number of participants meeting eligibility criteria, we estimate screening up to 1680 potential participants.
Inclusion criteria for women in this pilot study are as follows:
(i1) She is 18 or older.
(i2) She is currently supervised by a criminal justice entity, such as a community court or probation, parole or ATI.
(i3) She reports engaging in unprotected vaginal or anal sex with a male partner in the past 90 days
(i4) She reports any illicit drug use or binge drinking or in alcohol or drug treatment in the past 6 months.
In addition, potential participants will be ineligible if any of the following criteria are met:
(e1) Ability to speak and understand English is not sufficient to participate in assessments or intervention sessions.
(e2) The woman’s sexual activity is limited to a monogamous relationship lasting more than 12 months, and she has not engaged in any of the additional HIV risk behaviors in the past 90 days:

-Having sex with more than one partner
-Having sex with a partner known or suspected to be HIV positive or an injection drug user (IDU)
-Sharing injection drug use needles or equipment
(e3) The woman is actively trying to get pregnant/have a baby.
(e4) Inability to complete informed consent process due to a psychiatric or cognitive impairment.
(e5) The participant was born male.
The research staff who is administering the consent for screening and intervention will determine capacity to consent by asking comprehension questions such as “what are you agreeing to do by consenting to this research study?” and “how long will the screening interview take?” Additionally, research staff conducting will be trained to assess primary mental status, including orientation to person, place, and time, and signs of major cognitive impairment. In the end, if the research staff is not completely convinced that the participant can give full informed consent, the participant will be excluded from the study.

7. Recruitment

Active recruitment will occur on site at DOP probation offices and at parole or designated ATI program sites in NYC that are selected by DOP’s Deputy Commissioner for Adult Operations or his or her designee and the leadership of parole or designated ATI programs located in NYC. The Project Director will communicate as requested with DOP, parole or ATI program leadership personnel regarding recruitment strategies and will present a draft recruitment schedule to DOP’s Deputy Commissioner for Adult Operations or his or her designee, and representatives from parole or ATI programs in NYC for their review. The Project Coordinator will provide an invitation letter to DOP’s that contains the basic eligibility criteria and contact information of the study, to be send to their clients who might be a potential participant. Columbia research assistants, who have been certified in human subjects and HIPAA training, will recruit participants by approaching female clients after completing probation check in at kiosk stations, intake sessions or probation appointments and at parole or ATI program sites, and ask permission to talk to them about E-WORTH. We will also present the study to Probation, parole or ATI program staff.

If clients express interest, the research assistants will give them a project information flyer that provides a telephone number and basic information regarding the study. These flyers and other promotional materials must be reviewed and approved by DOP’s Deputy Commissioner for Adult Operations or his or her designee.

Recruitment will also occur via radio broadcasts on radio stations (David Rothernberg’s show on WBAI – Mr. Rothernberg’s is the founder of the Fortune Society, Unlocked with Ricky Jones on WHCR 90.3 FM – radio program targeting individuals involved in the criminal justice system) to which target population commonly listen.

RAs will introduce potential participants to the general nature of the study and other preliminary logistics (e.g., time, compensation). Clients will be informed that participation in the study is completely VOLUNTARY. If a participant chooses to participate or chooses not to participate, or chooses to participate and then decides not to participate, they will be advised that the study activities would not affect their status with the court, probation, parole or ATI programs or criminal justice system.

Passive recruitment will involve flyers and advertisements that may be posted or electronically delivered in locations in probation, parole or ATI program sites and community-based organizations that provide services to women on probation, parole or an ATI program.

In addition, the Project will play a video at Department of Probation, parole or ATI programs sites for the sole purposes of recruiting new participants in the study. The video will be played on DOP, parole or ATI program screens in areas in which women wait to be seen by probation, parole or an ATI program personnel.

In addition, study participants or women from probation, parole or an ATI program who screen ineligible will be asked to refer other potentially eligible women on probation, parole or an ATI program without revealing the exact eligibility criteria. Screened participants will also be asked to refer individuals to the study using a coupon referral system. We have successfully used these recruitment strategies in our past and current RCTs that we have conducted in probation.

Research staff will then ask the woman to meet in a private room to discuss screening procedures. This private room is in an area away from probation, parole or an ATI program staff and other clients. If the woman participant is not comfortable completing a face-to-face screening at the probation, parole or an ATI program office, research staff will arrange one of two alternate procedures: 1-the participant will be scheduled with a face-to-face screening interview at the Social Intervention Group research site, or 2-the participant will be instructed to call the study 1-800 number when she is in a private setting in order to be screened by phone. At the conclusion of screening, if the female on probation, parole or an ATI program meets eligibility criteria for the study and is willing to participate, a baseline assessment will be scheduled at the Probation, Parole or an ATI Program Site or the SIG Community Research Site. When a participant attends the baseline assessment, she will first be administered a brief confirmatory screener to re- establish major eligibility criteria (if the baseline occurs more than 24-hour after the initial screening). If the participant no longer meets eligibility criteria, she will be given $10 in compensation and will no longer participate in the study.

8. Informed Consent Process

8.1 Informed consent for participants for mainphase trial: If the participant still meets eligibility criteria, research staff will again describe the study, and the participant will be consented for participation in the study. The informed consent process will emphasize that involvement in research will include a baseline assessment, four intervention sessions, and three follow up assessments, for which the participant will be compensated. She will also be reassured that her decision not to participate or continue in the research study will not result in consequences from the court, NYC Department of Probation, Parole, ATI programs or the criminal justice system. If the participant agrees to take part in the study, she will sign the consent form and will be administered the baseline assessment and HIV/STI testing. After the baseline assessment and HIV/STI testing, the participant will be randomized into one of two conditions, and continue through the study as described in study procedures.

Research Assistants will go through the informed consent process with potential participants immediately prior to conducting the initial and the confirmatory screening interviews as well as

at the baseline and all follow-up CASI assessments. In addition, Research Assistants will administer the HIPAA consent form at the screening interview.

Obtaining informed consent will be documented with the use of a written consent form. The consent form provides details of the study procedures, risk, benefits, nature of confidentiality, study contact information, the nature of voluntary participation. The consent process also covers the information on reimbursement for time. Data will be destroyed with no questions asked upon a subject's request. The consent form will state that participants may withdraw from the study without penalty at any time for any reason with no explanation. Participants will also be asked about agreeing for the intervention session to be audio recorded solely for the purposes of quality assurance. The consent form indicates that such agreement is not a requirement to participate in the study and informs the participant that any portion or the entire recording will be erased upon her/his request at any time, including during or any time after the interview. Before a participant signs the informed consent form, staff will thoroughly review the form, ask if the participant understands the content of the consent form, and answer any questions that may arise. As noted above, informed consent will not be considered obtained (and thus, the participant excluded from the study) if a potential participant is unable to state the following after reviewing the consent form: (1) the nature and extent of participation in the study; (2) the risks involved with participation; and (3) the potential benefits of participation in the study. Informed consent for Probation, parole or an ATI program client participants for in-depth interviews: We will follow the general same consent procedures described above for participants who are randomly selected to complete a face-to-face in-depth interview. For randomly selected participants who express interest in completing an in-depth interview. Informed consent for Probation, parole or an ATI program staff and Fortune providers for Aim 4 study activities: For probation, parole or an ATI program staff and providers who express interest in being interviewed in the longitudinal survey, research assistants will similarly go through the informed consent process in a private location and ensure participation is entirely voluntary, stressing that participation is in no way required for employment purposes, and their employer will not know whether or not they chose to participate.

9. Confidentiality of Study Data

All data are collected specifically and only for the proposed study. Data management activities and procedures will utilize the electronic data management systems used in all recent and current SIG studies. These protocols were designed to enhance the efficiency, security, and integrity of study data. This system includes: (1) secure and confidential WWW-based (a) scheduling information for RAs , (b) automatically generated summaries to tailor recruitment efforts, (c) uploading of digital audio recordings for QA protocols, and (d) collection, monitoring, and summary reporting of data relevant to day-to-day operations of the study (e.g., petty cash disbursements); and (2) custom-programmed data entry software to ensure consistency, integrity, and security/confidentiality of the transfer of written information into electronic databases. Additional procedures are described below. Data collected using CASI during baseline and follow-up assessments are automatically recorded/stored by the computer (i.e., no hardcopy of the data is generated). The study will use DatStat products and services for creation and administration of CASI as well as management/transfer of CASI data. DatStat data security meets strict government regulatory compliance. DatStat delivers SSL (Secure Socket Layers) and our secure key is provided by VeriSign. As respondents move through the assessment, responses are encrypted for transit between the respondent's browser and the DatStat server using SSL and

128-bit encryption. DatStat is used by researchers across the country and is very familiar with strict research methodology, security needs, and requirements of/responsiveness to human subject protection, IRB, and HIPAA issues.

Digital recordings of in-depth interviews, in-depth interview transcripts and survey data from the CASI interview with Fortune providers will be assigned a study identifier code. Only the PI and the Project Director will have password protected access of the encrypted file linking names of Fortune provider participants and their study identifier code. Participants will be assigned a unique identifier code that is different from their study identifier code to log into the multimedia web program. The file linking the web program identifier and the study identifier will only be accessible to the PI and the Project Director. The web program will only collect usage data regarding the resources in the program. The program will not collect personal identifying information such as email addresses, usernames, IP addresses, or names. Usage data that are collected by the web program will be password protected and accessible only to the PI and the Project Director.

The Project Director will post recruitment/interview schedules for RAs on an encrypted password protected web page dedicated to the study. At the end of each day, RAs complete a daily, on-line summary form which includes data entry fields concerned with operational data (e.g., number of potential participants approached, number and reason(s) for refusal). Although the dedicated web page is password protected, no identifying information is posted/submitted by research staff because web server resources rely on Columbia University technical staff members who are not involved with the study as well as the frequency of security threats discovered by manufacturers of common web browsers (e.g., Microsoft). However, we prefer to use this WWW interface because summary reports (e.g., recruitment rates by RA, by study site, etc.) can and will be automatically generated and e-mailed weekly to the PI and Project Director. These procedures have been implemented without incident by the SIG investigative team in other studies. Study staff will also use the internet to transfer and securely store digital audio recordings used for quality assurance purposes. Digital recordings are transferred and stored on a dedicated FTP server maintained by and accessible only to SIG staff. Centrally storing all recordings on a password- and firewall-protected computer enhances security (e.g., all attempted accesses, whether successful or unsuccessful, are automatically logged and reviewed weekly by a dedicated SIG computer support staff member) and integrity (e.g., automatic backup of recordings using 128-bit AES encryption onto optical media). These procedures were developed by Dr. Wu and currently in use for all current SIG studies. At the completion of the study, all digital files with identifying information—including audio recordings—will be destroyed by using software that meets DoD 5220.22-M specifications for files on magnetic media, overwriting files stored on the flash memory used by digital recorders, and physically shredded for files stored on CD/DVD media.

Safeguarding the confidentiality of personal data reported in verbal questionnaires or during other data collection/documentation (e.g., HIV test results) is maintained through a tear sheet procedure; the participant's name is removed from the forms after initial checking, leaving only an identification number, which ensures the confidentiality of the data throughout the study. All interview data are coded to preclude participant identification; no personal identifiers as defined by the Health Insurance Protection Accountability Act (HIPAA) will be included in screening or assessment data instruments with participants and Probation, parole or an ATI program staff and Fortune providers. Research staff will verify that all screening instruments

are gathered before they leave the data collection site. All data from screenings will be coded and entered into a database. All data files will be encoded using 128-bit AES encryption and password-protected on end computer devices with both hardware and software firewalls. Electronic sensitive study data will also be stored on Columbia University's secure and HIPAA approved Courseworks multi-user system site.

In addition, the informed consent, locator form and any signed paperwork are kept in a separate folder and to not contain the research identification number. A name/code number roster will be kept locked in a file cabinet separate from the assessments. All research forms and instruments are stored in locked file cabinets in a private office in an access-controlled building. Additionally, Dr. Gilbert has secured a Certificate of Confidentiality from the NIH to avoid subpoena regarding confidential information from subjects. Any attempts to subpoena research information will be immediately reported as an adverse event to the principal investigator and the IRB.

10. Privacy Protections

All face-to-face screening interviews and face-to-face in-depth interviews will take place in a private room either at the probation, parole or an ATI program site, a Fortune site or at our community research site.

All ACASI (Audio Computer-Assisted Self Interview) interviews will take place in an enclosed cubby space or kiosk with head phones so that others may not hear questions or view responses. Although some research activities may take place within a criminal justice setting, all information collected in the interview is confidential, and will not be shared with any criminal justice entity. This includes information about illegal activity, such as drug use. Probation, parole or ATI program department personnel have affirmed that they will not seek identifiable information on research participants. The only instance where research staff are required to release identifiable information is in the case of imminent harm to yourself or others, or if there is a report of child abuse or neglect. We have obtained an assurance letter from Fortune Society andMemorandum of Understandings (MOUs) from the NYC Departments of Probation, Parole and designated ATI programs providing assurance that they will not seek the data collected about subjects. The general letter of introduction will be composed for the intervention team to introduce them to the supervisors of each probation, parole or an ATI program site where recruitment is to occur, with instructions to provide a copy of the letter to all probation, parole or ATI program officers at each such site. The researchers have taken steps to assure privacy and the confidentiality of the information provided by the participants. These steps include obtaining a Certificate of Confidentiality and the assurance of Fortune Society that it will not disclose any information to others or seek access to it. Further, probation, parole or ATI program sites will not have any documents linking the participants name to any information given to the researchers. In the criminal justice system, however, there are no absolute guarantees of confidentiality. If participants have signed a waiver of confidentiality or release (or sign one in the future), the holder of such a waiver or release may seek to obtain information from the researchers. The researchers will resist disclosure of such information by all lawful means.

All study staff will undergo a common training that includes the impetus, goals, organization, and rules/regulations under which the study operates; this common "base" training also covers:

(1) ethical issues in research; (2) establishing and maintaining rapport with participants; (3) obtaining informed consent; (4) addressing participants' concerns with confidentiality and handling sensitive situations; (5) obtaining accurate tracking information; (6) managing distress and conducting crisis and enhanced referrals; and (7) detecting, handling, and reporting adverse events. This common, general training is supplemented with more specialized topics and scenarios during trainings dedicated to specific roles/tasks (e.g., Fortune providers or RAs who deliver HIV/STI results) as appropriate.

Confidentiality of in-depth interviews with Probation, Parole or ATI program staff and Fortune providers: In addition, for the purposes of improving this program, participants will be audio taped in the sessions and/or audio taped during an interview session. Only research staff will review these tapes. The tapes are for research and training purposes only, and will be erased after the study is completed. Interviewing records will only be shared with the research team. Recordings will be downloaded to a secure and password protected for storage and then erased from the digital recorder. The recording will be reviewed by a research assistant for accuracy and thematic analysis. Any identifying from the defined by the Health Insurance and Portability Act (HIPAA) will be immediately deleted from the recording. All digital recordings will be destroyed at the end of the study after they have been transcribed and the transcriptions have been checked for accuracy. All electronic files transcripts and thematic analyses of transcripts will be encrypted and password protected.

Disclosure Protocol: By virtue of the study population, it is anticipated that some of the subjects may disclose past or current engagement in law breaking activity (e.g., sex trading, selling drugs, theft, etc). Following protocols used in the pilot study and prior SIG research with drug-involved offenders, research staff and Fortune providers will inform participants of the limits of confidentiality at the start of session. Research staff will not release any information about participant to anyone unless there is a possibility of "imminent harm to self or others" and/or, through group or individual discussions it becomes clear that a child is in danger or is experiencing physical abuse or neglect. Dr. Gilbert has secured a Certificate of Confidentiality from DHHS to avoid subpoena regarding confidential information
from subjects upon receipt of funding for this study.

Suicidal and Homicidal Respondents: Confidentiality cannot be maintained if a participant is homicidal or demonstrates intent on seriously injuring another person. Similarly, a respondent may indicate that he is considering hurting himself. Following protocols used in prior SIG studies intervention trials with populations at elevated risk for HIV/STI transmission, we will inform the appropriate agency (e.g., law enforcement, hospital) in these cases. Research assistants will be trained to handle either situation in the event it occurs, and these procedures will be described in the informed consent forms and reviewed with all participants during consent procedures. Like the procedures for referrals, data regarding implementation of procedures to address suicidal/homicidal participants will be collected and analyzed in a parallel manner to investigate the effects of the research process itself on utilization/involvement with the service system and other outcomes.

Child Abuse/Neglect: By law, the privilege of confidentiality does not extend to information about the sexual or physical abuse or neglect of a child. If a participant makes statements from his personal knowledge, which if correct, would render a child abused or neglected, New York City's Administration for Children's Services (ACS) will be notified. The PI will make every

effort to attempt to ensure the safety of participants. Participants will be informed of these mandates during administration of the informed consent protocol. Like the procedures for referrals and suicidal/homicidal participants, data regarding implementation of procedures to address child abuse/neglect will be collected and analyzed in a parallel manner to investigate the effects of the research process itself on utilization/involvement with the service system and other outcomes.

11. Potential Risks

Screening, assessments, and interventions should pose minimal risks to subjects participating in the study. Subjects may become embarrassed or uncomfortable while being asked questions and there is some risk that discussing sensitive topics will cause distress. Because subjects will take part in a group intervention, there is a risk that other group members may divulge confidential information to people outside the group. We will address this by asking everyone to respect the confidentiality of the other participants. Participants will be advised that conversations about HIV and safer sex negotiation may anger their male partners. This anger may put them at risk for emotional, physical and/or sexual abuse or abandonment of the relationship. Participants will be advised to observe any signs of such anger in their partners and to discuss them with study staff immediately.

Study staff will be trained to provide crisis intervention and referral for such situations as follows: All project staff will be trained to observe verbal and non-verbal signs that may indicate that the respondent is emotionally distressed. In cases where the respondent is exhibiting distress, the data collector, recruiter, or facilitator will acknowledge that the questionnaire or group session can raise troubling issues for respondents and ask the respondent how she is feeling and whether or not she wants to take a break from the interview/group. If the respondent continues to exhibit distress, the data collector/recruiter/facilitators will ask her if she wants to terminate the interview or session and encourage her to discuss the issues further with any existing counselor. If a respondent seems unresponsive or unwilling to go to the counselor, or if there is no current counselor, the researcher staff will offer her a range of counseling options in the local community, using a resource manual developed for this project that contains local services and resources with upto-date contacts who have indicated a willingness to serve women from our study population. Project staff will refer participants who request such services to professionals who we have identified in the community. After assisting the participant in exploring the counseling, residential and/or legal services available, the facilitator will offer to assist the participant in locating appropriate services and will provide enhanced referrals (e.g., calling the organization and assisting in making an appointment). If the participant is undecided about needing help and is not ready to take a referral, the facilitator will give the participant a business card with the phone number of the Project Director and encourage her to call if she would like a referral in the future. Acute emergency distress referrals will be directed to the designated counselor or social worker, and the Project Director and Dr. Gilbert, who is a certified social worker, will be alerted. In the event that a respondent indicates to research staff that she is suicidal or homicidal, a designated clinic counselor or a SIG certified mental health professional will be informed and available for crisis intervention. At the end of all assessment and intervention sessions, whether or not complete, staff will ask anxious or troubled participants for permission to discuss their discomfort with their treatment program counselors. If permission is granted, research staff will walk distressed participants directly to the counselor. Any participant choosing not to be referred to their counselor will receive counseling from trained

certified professional mental health counselors on the research team, including the PI, Co-Investigators, and the Project Director, available as back-up to treat and make enhanced referrals for distressed participants.

Additionally, risks from this project may arise from obtaining specimens, or from conducting a rapid oral HIV test. Women may feel uncomfortable providing specimens for STI testing, providing an oral swab to test for HIV, or receiving pre- and post-test counseling for HIV. This project also involves risks that are associated with positive STI and HIV testing. The possible diagnosis of an STI or HIV and partner notification could cause emotional distress. Although unlikely, breach of confidentiality regarding STI status, HIV serostatus, sexual behaviors and relationships resulting from participation in this intervention may cause participants some level of psychological, social or financial distress. There is a possibility of unauthorized disclosure of sensitive information shared during screening, intervention sessions, or assessments. To minimize the likelihood of unintended disclosure and to maintain confidentiality safeguards, research staff will be trained to remind participants that they may at any point request their data be destroyed without question. Additionally, the NYC Departments of Probation, Parole and designated ATI program sites have indicated in letter of supports that they will not request any participant information obtained through the study. By virtue of the study population, it is anticipated that some of the subjects may disclose past or current illegal activity (e.g. sex trading, drug activity, theft, etc.). Other possible types of information disclosed are intimate partner violence, suicidal or homicidal ideation, or
child abuse/neglect. Following protocols used in prior SIG research with drug-involved offenders, research staff will not release any information about participants to anyone unless there is a possibility of imminent harm to self or others and/or actual or suspected child abuse or neglect.

11.1 Vulnerable Populations

The proposed study will not involve participants who meet the criteria for classification as prisoners. As the study will involve participants who are in probation, parole or ATI programs, there is a likelihood that some participants may become incarcerated. If a participant becomes incarcerated or detained, the adverse events protocol described below will be followed, and the participant's involvement in the study will be discontinued.

12. Adverse events, Protocol Violations and Procedural Variations

A policies and procedures manual for the project will specify immediate data monitoring and response for anticipated health or mental health issues. Timely data analysis will ascertain if addressable prevalent conditions occur. Data on adverse events and psychological distress will be systematically collected during the trial and reported to the PIs and the Columbia University IRB in order to secure appropriate management of the adverse events as well as to have full documentation of the events for all conditions. The following steps detailed below will be used. All research staff will have completed the Columbia University Office of Projects and Grants' Human Subjects training course, which has received approval from DHHS Office of Human Research Protections to supersede the NIH training course on Protection of Human Research Subjects. The Columbia University training also includes compliance with the Health Insurance Portability and Accountability Act of 1996 (HIPAA). As part of SIG's standard operating protocols, all SIG field, quality assurance, and management staff must complete an intensive,

day-long, structured training program on detecting, addressing, and reporting adverse events before they are allowed to participate in any research. This training which will be conducted by the PIs will also cover how to handle challenging situations, including how to respond to distressed participants and participants who are experiencing life threatening IPV. Also, each research study at SIG conducts an annual 3-hour meeting, first reviewing human subjects protection principles and study-specific procedures, followed by discussion of adverse events for the study over the past year, and role-play exercises which provide a booster to enable staff to handle future adverse events knowledgeably, appropriately, and expeditiously.

Serious adverse events include death, a life-threatening event, an event resulting in hospitalization, prolongation of hospitalization, disability, and incarceration. Serious adverse events, regardless of whether or not they are study related, will be reported to the Columbia University IRB. Other adverse events include breaches of confidentiality (both intentional - e.g., mandated reporting of suicidal/homicidal participants - and unintentional), and non-life threatening psychological distress. The PIs will closely monitor adverse events. The investigative team is aware that the study sample is likely to experience a relatively high incidence of adverse events due to their past/current history of law-breaking behavior, rates of recidivism/re-incarceration reported in the literature, and the high levels of service needs noted earlier in this application. Drawing upon past and ongoing SIG studies, the investigative team designed two forms, (1) Adverse Event: Initial Report and (2) Adverse Event: Full Report, to document adverse events, staff responses, and disposition. The procedures used to track, report, and examine adverse events are described below.

Protocol to Handle Each Adverse Event: Study staff and Fortune providers will identify, manage and document all adverse events. These events may be identified by RAs, the Project Director, PI, or other project staff, including quality assurance staff reviewing audio recordings of interviews, or they may be reported by participants. The following steps will be used to monitor, track, and document procedures used to address each adverse event and subsequent disposition. Within 12 hours of an adverse event being reported/detected, an Adverse Event: Initial Report study report form will be completed by the study staff member or Fortune provider who identified the event. The report form will include date, description of the event, duration, severity, measures taken to ameliorate the adverse event, and disposition-related information (e.g., time spent providing referrals and type of referral (domestic violence shelter, mental health or substance abuse treatment, etc.). The report form will be reviewed and signed by the immediate supervisor who will be responsible for ensuring that appropriate actions have been taken. Within 24 hours of the adverse event, the research staff member or provider who identified the adverse event will discuss the event and response with the Project Director (or the PIs if the Project Director is unavailable) in order to ensure adherence to the protocol for handling the event. (3) Within 48 hours of the adverse event, the investigative team, the Project Director, and any involved staff will ascertain whether the event was related to participation in the study.

An Adverse Event Full Report form will be completed by Drs. Gilbert and El-Bassel. This full report will contain an attached copy of the initial adverse event form, a summary of information obtained from other documentation and sources that clarify the nature of the event and outcomes, and the determination of whether the event was study related and by what criteria such determination was made. (4) Drs. Gilbert and El-Bassel will be responsible for reviewing the event occurrence with the appropriate staff to ensure that an adequate response is provided to the

participant. Any serious adverse event whether or not related to the study will be reported to the Columbia University, and the NIDA project officer for the study. The initial adverse event report will be submitted to Columbia University's IRB. This will be followed by submission of the full report to the IRB. In the event that a participant withdraws from the study or the investigator decides to discontinue a participant due to an adverse event, the participant will be monitored by the investigators via ongoing status assessment until (a) a resolution is reached, (i.e. a problem requiring hospitalization is stabilized with no further change expected, or (b) the event is determined to be unrelated to the study.

Oversight and Review of Adverse Events: Every two months, the entire investigative team will review all adverse event data to date to determine if systematic trends exist among adverse event data to warrant changes to study protocols and procedures. Any proposed changes will be reviewed with staff at the study sites, and, if needed, external consultant (e. g., other senior investigators conducting federally-funded research on drug abuse and/or criminal justice). The investigative team will also conduct systematic analyses of adverse event data (e.g., associations among frequency, type, severity of adverse events; participant characteristics; and operational aspects of research such as time point, gender of interviewer) to inform future studies with participants in probation, parole or ATI programs as well as recognizing such activities represent a potentially under- researched area of inquiry and scientific endeavor unto itself.

Protocol Violations and Procedural Variations – protocol violations and procedural variations from established study protocols and procedures will also be documented by the Project Director and the PI and reported to the IRB using the Protocol Violation and Procedural Variation Forms. A binder of procedural variations will also be maintained by the Project Director.

13. Data Safety and Monitoring

The Data Safety and Monitoring Board (DSMB) will oversee and provide advice to the PIs on the continued scientific integrity and safety of the implementation of the trial. At each meeting the board will review the status of recruitment, retention, successes, barriers and challenges of the implementation of the trial, adverse events and the reporting procedures of adverse events. Two interim analyses will be conducted in addition to the planned "final" analyses at the conclusion of the proposed study. These interim analyses will be conducted when 33.3% and 66.7% of the planned sample size have passed their 12-month follow-up assessment time point window. The DSMB will review findings from the interim analysis and determine whether the study needs to be terminated for safety reasons. Data will be sent to the DSMB a week prior to the meeting. The Board will meet twice a year. The meetings will be face-to-face. Minutes taken at the meeting will be archived. We have included a statement in the DSMP that the proposed Board members have no Conflict of Interest with the study. The DSMB will consist of four expert members in the field who are experienced with HIV treatment, substance abuse treatment, and vulnerable populations. The DSMB will meet twice a year. The DSMB will consist of three expert members in the field who are experienced with HIV treatment, substance abuse treatment, and vulnerable populations. Specifically the members will include:

1. Wendee Wechsberg, PhD, is the Senior Director of the Substance Abuse Treatment Evaluations and Interventions Research Program at RTI International. Since its inception in 1999, this program has evolved to include a multimillion dollar yearly portfolio. She is also

Adjunct Professor at the School of Public Health at UNC, Chapel Hill, and Adjunct Associate Professor of Psychiatry at Duke University School of Medicine. She has conducted multiple NIH-funded clinical trials testing gender specific HIV interventions with different populations of women drug users.
2. Sandro Galea, MD, is a Professor of Epidemiology and Public Health at Columbia University School of Public Health. He is an expert in addiction medicine and HIV care among drug users. Scarlett Bellamy, PhD is an Associate Professor of Biostatistics at University of Pennsylvania. She is an expert in developing data analytic plans and analyzing outcome data for clinical trials and will perform the interim analyses.
3. Jack DeHovitz, MD is a Professor of Medicine at SUNY Downstate Medical Center. He has extensive experience conducting clinical research with HIV-infected women drug users.

Interim Analyses and Stopping Rules: Two interim analyses will be conducted in addition to the planned final analyses at the conclusion of the proposed study. These interim analyses will be conducted when 33.3% and 66.7% of the planned sample size (N=140 and N=280 women respectively) have passed their 12-month follow-up assessment time point window. Several considerations and/or constraints were balanced in deciding upon key aspects of interim analyses. We restricted the number of interim analyses to 2 in order to prevent too great a threat to valid statistical inference due to a theoretical need to adjust analyses to avoid Type I error inflation from multiple comparisons using the same data. Interim analyses will be conducted at the selected information rates (i.e., the 33.3% and 66.7% values) based on 12-month follow-up duration period as per recommendations in Methodological Challenges in Biomedical HIV Prevention Trials published in 2008 by the Institute of Medicine (IOM). Specifically, in the chapter entitled Interim Monitoring and Analysis of Results, the IOM recommended that stopping HIV prevention trials based on positive interim results should require evidence of a sustained impact. Thus, interim analyses will utilize complete data over the longest follow-up duration, i.e., the 12-month assessment time point. The Principal Investigators and Co-Investigators will be unblinded to the research, whereas the study staff will be blinded to the interim analysis. The interim analysis will be conducted by the statistician on the study and the results will be reported to the PIs. With respect to efficacy, stopping rules following an interim analysis focus on demonstrable benefit among the sample and data accrued to date.
At each interim time point as well as after study completion, analyses will follow the data analysis plans for primary aims described in the original proposal. Interim analyses will focus on the study's primary aims. If at any of the interim analyses the statistical significance for all biological outcomes related to primary aims (i.e., cumulative STI incidence), exceeds the a prior a < .05 criterion at that time point, the study will be stopped since the inference will be that significant benefit of assignment to one study arm over the other will have already been demonstrated. We note that a represents two-sided hypothesis testing since demonstrating benefit with respect to disease prevention of one intervention over the other (regardless of the content of that intervention) is of public health value and importance. With respect to futility, stopping rules are concerned with effect size estimates indicating a null result is inevitable given the planned total sample size. Stopping a study in such circumstances avoids additional expenditure of time, fiscal, and human resources that would nevertheless ultimately result in lack of evidence of superiority of one intervention over the other. At each interim analysis time point, a post hoc power analyses will be conducted to determine the minimum sample size needed to achieve 80% power to reject the null hypothesis given point estimates and standard errors of efficacy estimates using data collected to date. If the estimated minimum sample size for power analyses using a =

.05 exceeds the planned enrollment of N=420 women for all of the biological and self-reported behavioral outcomes associated with the primary aims, the investigative team will notify the NIDA Program Official (PO) of such findings. Based on the magnitude of the difference between the estimated minimum sample sizes needed for 80% power and targeted sample size, in addition to recruitment rates to date, the investigative time and NIDA PO will discuss and agree whether the study should be stopped due to reasons of futility or whether modifications to the study design/protocols (e.g., increasing planned enrollment, hiring of additional recruitment staff, etc.) can be implemented to avoid futility.

14. Potential Benefits

Potential benefits to study participants are learning skills and information that may lead to decreased risk of contracting and transmitting HIV and other STIs. The benefit to society is that the investigators may develop a better understanding of which interventions are most useful for promoting safer sex among justice-involved women.

15. Alternatives

N/A

16. Research at External Sites

The investigative team has conducted several randomized controlled trials (RCTs and prevention and services research in criminal justice systems, including three recent or current RCTS with the NYC Department of Probation. They have established strong collaboration with the NYC New York City Department of Probation Sites: Participants will be recruited from probation sites throughout NYC. We will also recruit participants from parole and designated ATI program sites. We will particularly target sites that serve low income minority communities in NYC most heavily affected by HIV/STIs. The investigative team has collaborated with Probation on three HIV research projects since 2008. Projected Enrollment: In 2012 there were at total of 3,115 Black women at the 5 DOP sites. Given the annual pool of Black women on probation from the five study sites (N=3,115) as of January 2012 and the given estimated number of 1,250-1500 additional Black women per year who will be placed on probation during the enrollment period at the 5 sites (N=3,993), we project a total recruitment pool of 7,045 Black women on probation specifically who may be approached for screening. Of this pool, we estimate 70% (N=5,607) will agree to complete a screening interview based on our current and recently completed studies in probation of whom 35% (1,726) will meet eligibility criteria based on studies summarized earlier and enroll in the study. This number will augmented by women on parole or an ATI program. This flow of potential participants will be ample to achieve a sample size of 420 women.

Staff from Fortune Society, which is the largest probation contract provider in NYC, and also provides services to individuals on parole and other ATI programs, will be delivering both interventions for this study (see letter of support) either at their site or at one of the probation, parole or ATI program sites. Fortune Society does not have an IRB or access to an IRB. Stanley Richards, our administrative contact at Fortune Society, has indicated that they will accept Columbia as the Sole IRB for review and approval of this study. The NYC Departments of Probation and Parole, and leadership personnel from designated ATI program locations, will

provide space for recruiting participants and conducting assessments at probation, parole and ATI program sites located throughout in New York City, however, they will not have any contact with human subjects for this study. New York City (NYC) Department of Probation, Parole and ATI Sites: In New York State, probation, parole or ATI are court-ordered sentences to community supervision with certain conditions. NYC operates probation and parole departments, and ATI programs, with funding and oversight by the New York State Division of Probation and Correctional Administration (DPCA). Each of the five NYC boroughs has probation, parole and ATI program reporting sites, where offenders under supervision report to probation, parole and ATI program officers with frequencies ranging from weekly to several times a year.

Social Intervention Group- Community Research Site: The Social Intervention Group (SIG) at the Columbia University School of Social Work (CUSSW) has a new community space where we conduct all assessments and intervention sessions, which is located within a 10-minute walk of our administrative offices at CUSSW. This community space was outfitted for this study and contains two gender specific assessment rooms that are equipped to conduct the Audio Computerized Assisted Structured Interviews (ACASI), a bio-specimen lab, which is equipped to process, store and ship specimens; four private offices where the face-to-face interviews and pretest and post-test HIV/STI counseling are conducted; four intervention rooms – one of which is outfitted with PCs to conduct Web based multimedia interventions. These intervention rooms can comfortably fit up to 14 people and are equipped with the audiovisual equipment, flip charts and all other materials specific to each intervention. This community space also has two office rooms with workstations to accommodate project staff while they are in between sessions and locked file cabinets to store confidential materials.

17. Columbia as Lead Institution

Columbia University is the lead and sole institution implementing this project. Letters of support from Fortune Society and the New York City Departments of Probation and Parole, and designated ATI program locations are attached. As Fortune Society does not have an IRB, we applied for and received IRB coverage through Columbia University. We have also secured an IIA Agreement (FWA00022811).

18. **E-WORTH Study protocol to allow multi-level statistical analysis**

The following describes additional research procedures at to accommodate a spatial multi-level analysis of the data that incorporates neighborhood aggregate data in the statistical analyses of these data. Multi-level statistica analyses will examine the association between socio-contextual variables aggregated at the Community District and United Hospital Fund Level and outcomes of sexually transmitted infections. In order to carry out the proposed research it is necessary to attach an additional variable indicating which Community District and United Hospital Fund neighborhood corresponds to each participant. The following modification describes necessary research procedures and safeguards to protect the confidentiality and privacy of all study participants during the spatial analysis process.

Research procedures

All participants with addresses will be geocoded using QGIS software on a secure computer at the research site. Once addresses are geocoded using the spatial merging options available in QGIS the geocoded addresses will be attached to another spatial shapefile that indicates corresponding community districts and United Hospital Fund Neighborhoods. The resulting shapefile will be exported into a STATA file and the column variable

indicating addresses of participants will be deleted prior to removing the file from the secure computer at the research site for statistical analysis.

Variables that will be used in this analysis

Aggregate variables that will be used in this study include the following: HIV incidence, % uninsured, unmet medical needs, alcohol use, heroin/prescription pain medication use, sociodemographic factors that include race ethnicity, poverty, homelessness, crime complaints offense data, and arrest data. Aggregate neighborhood level sexual risk variables from the publicly available Community Health Survey include, sex without a condom and number of sexual partners. All aggregate data is fully de-identified by government agencies prior to public distribution. This study will not analyze any identifiable aggregate data in this multilevel analysis.

Protection of human subjects

The proposed research will examine the effects of neighborhood contextual factors on sexually transmitted infections and other outcomes among participants in community corrections in New York City. In addition to the measures taken to protect human subjects in the approved protocol, participants who live in either community districts or United Hospital funds with cell sizes of 5 or fewer will be 'suppressed' or excluded from the final dataset. No addresses will be exported in the final analytic datafile. No maps will be created visualizing the data from the E-WORTH Study. Maps will be created visualizing aggregate data for contextual variables in New York City.

Geographic units of analysis

The two Aerial Units of analysis in this study are community districts and United Hospital Fund neighborhoods ***Community Districts.*** There are a total of 55 community districts in New York City with corresponding community boards that advise the local borough president on city budgeting of financial resources and service delivery in each of the districts. The districts are meaningful for public policy and public health in New York City. **United Hospital Fund** The United Hospital Fund districts consist of 34 neighborhoods in New York City The United Hospital Fund is a philanthropic organization that conducts nonprofit health service delivery research in New York City. The UHF neighborhoods provide geographic boundaries for illustrating the geographic distribution of many communicable and non-communicable diseases, social risk factors, STIs, and other variables.

Table 1. Aggregate variables that will be used in this analysis

Variable	Year	Source
Population by Neighborhood	2010	American Community Survey (Census Bureau)
HIV/AIDS overall by sex and race/ethnicity	2013	New York City Department of Health and Mental Hygeine
% Uninsured	2013	Epiquery Dashboard Community Health Survey
% with unmet medical needs	2013	Epiquery Dashboard Community Health Survey
Binge drinking	2013	Epiquery Dashboard Community Health Survey
Heavy drinking	2013	Epiquery Dashboard Community Health Survey
Heroin use	2013	Epiquery Dashboard Community Health Survey
% Black	2010	American community survey (Census Bureau)
% Hispanic	2010	American Community Survey (Census Bureau)

% White	2010	American Community Survey (Census Bureau)
% Asian	2010	American Community Survey (Census Bureau)
% Other	2010	American Community Survey (Census Bureau)
% felt unsafe in neighborhood	2016	American Community Survey (Census Bureau)
Crime complaints	2016	New York Police Department
Number of sexual partners	2016	Epiquery Dashboard Community Health Survey
Social indicators	2010	NYC open data
Rates of Crime	2014	

Data Analyses Plan:

The analytic approach is built upon the investigative team's prior HIV prevention randomized clinical trials, including an intention-to-treat approach, handling missing data using both complete case and multiple imputation approaches, attrition analyses to investigate non-ignorable nonresponse, and sensitivity analyses to ascertain robustness of findings against violation of model assumptions (e.g., distribution requirements, heteroscedasticity, independence). Mixed-Effects Generalized Linear Models will be used to account for the non-independence in measures due to repeated measures with the same person. These models build upon $g(E[(Y_{ij}|\mathbf{x}_{ij})]) = \mathbf{x}'_{ij}\boldsymbol{\beta}$ where Y_{ij} is the outcome variable of interest for the ith participant at the jth timepoint. The components of $\boldsymbol{\beta}$ will be intervention assignment, baseline attributes for the outcome variable, and selected sociodemographic variables based on the unbalanced randomization or relevant literature. The $g(a)$ function represents a link function that can properly model the outcome variable as a function of measurement unit or distribution. Hypothesis testing follows the form "Assignment to E-WORTH will be associated with significantly lower/higher on outcome variable Y at the 12-month follow-up or over the follow up period compared to assignment to the control condition where 'lower' or 'higher' is chosen in the direction of reduced risk. To test the difference in the new incidence of any STI (primary outcome) at the 12-month follow up, logistic regression models will be used. To test the effects for behavioral outcomes over the entire follow-up period, mixed-effects Poisson or negative binomial regression will be used for the number of condomless acts of sex (primary outcome), mixed-effects linear regression will be used for proportion of protected acts of intercourse, and mixed-effects logistic regression models will be used for binary behavioral outcomes.

Columbia University Human Subjects Study Description Data

Sheet

Protocol: IRB-AAAN8409 (Y1M00) Protocol Status: Approved Effective Date: 03/28/2018
Expiration Date: 03/27/2019
Originating Department: SSW Social Intervention Group (576000X)
Submitting to: Morningside
Title: Multimedia HIV/STI prevention for Black Drug-involved women in community corrections
Sponsor Protocol Version#:
Abbreviated title: Multimedia WORTH with MM-WORTH -Black Drug-involved women in community corrections
IRB of record: Columbia University Morningside
IRB number used by the
IRB of record:
Protocol Begin Date: 07/01/2014
Protocol End Date: 06/30/2019
Previous Protocol Number: IRB-AAAD5608
07/23/2015
Principal Investigator: Louisa Gilbert (571000X)

Study Description

1. Study Purpose and Rationale

1.1 Purpose: The proposed study is a randomized controlled trial (RCT) that will rigorously evaluate the effectiveness and cost-effectiveness of delivering a multimedia evidence-based intervention (E-WORTH which is an acronym for Empowering African-American Women on the Road to Health) and streamlined HIV testing to prevent HIV and other sexually transmitted infections (STIs) with Black/African-American (hereafter referred to as Black) women drug users in probation, parole or alternative to incarceration programs(ATI hereafter) sites in New York City (NYC), compared to streamlined HIV testing alone. Repeated assessments will occur at baseline and 3, 6, and 12-months post intervention. The primary outcome will be to reduce cumulative incidence of biologically confirmed STIs (i.e., Chlamydia, gonorrhea and trichomonas. This study has the following specific aims:

1.2. Primary Aims
1. To test the comparative effectiveness of E-WORTH versus Streamlined HIV Testing on primary outcomes of decreasing biologically confirmed STIs and the number and proportion of unprotected sexual acts at the 12-month follow-up and secondary outcomes of reducing drug use, increasing utilization of drug treatment, linkage to HIV care and ART adherence (for HIV+ participants), decreasing partner violence and recidivism.
2. To test if the effectiveness of E-WORTH on study outcomes is moderated by client characteristics (e.g. client subgroups defined by socio-demographics and psychosocial characteristics)
3. To estimate the costs and comparative cost-effectiveness of E-WORTH versus Streamlined HIV Testing on STI infection rates, drug use and projected number of HIV cases averted at the 12-month follow-up.

4. To examine qualitatively and quantitatively how multi-level theory-driven factors may influence the fidelity of implementation and effectiveness of E-WORTH and Streamlined HIV testing on study outcomes.

1.3 Rationale: Despite extremely high rates of HIV and STIs that have been found among women on probation, parole or ATI programs nationwide, to date, WORTH is the only intervention that has been found to be efficacious in increasing condom use and reducing sexual risks among justice involved women in the U.S. Our recent study of 337 drug-involved women on probation or community supervision in NYC found that 17% of the 221 Black women tested positive for HIV and 30% tested positive for an STI 3. The HIV rate found among Black women on probation indicates a highly concentrated epidemic, comparable to rates in sub-Saharan Africa; however, no HIV prevention or testing services are provided in NYC probation sites. The proposed study will address this critical gap in services for Black women drug users in probation settings by testing the effectiveness and cost-effectiveness of the 5-session multimedia E-WORTH intervention.

2. Study Design and Statistical Procedures

2.1. Overview of Study Design: This RCT will be conducted with 420 drug-involved Black women recruited from probation, parole or designated ATI program sites in NYC. Women will be randomly assigned to either (1) the 5-session E-WORTH intervention condition that includes an individual Streamlined HIV Testing session or (2) a Streamlined HIV Testing session (See Section
C.9). Fortune providers will deliver both intervention conditions at the Fortune sites in Long Island City or Castle Garden in Manhattan. In extenuating circumstances (e.g. participants are located too far away from these sites), Fortune providers may also deliver both conditions at a NYC DOP sites approved by the appropriate DOP Assistant Commissioner or at our community research office. Women assigned to either condition will continue to receive all services provided by probation, parole or designated ATI program staff. Women who test positive for HIV in either condition will receive HIV post-test counseling and linkage to treatment using the CDC Counseling, Testing and Referral (CTR) Protocol. In month 14, recruitment of participants will begin. We will enroll an average of 14 women per month and complete enrollment by month 45. Self-reported data on behavioral outcomes will be collected from participants via audio computer-assisted self-interview (ACASI). The primary outcome will be measured via biological assay for infection by Neisseria gonorrhea, Trichomonas, and Chlamydia trachomatis, the most common STIs found among women in NYC.

Repeated measurements will take place at baseline and at 3, 6, and 12 months' post-intervention. For Aim 3, we will collect cost data from a variety of sources every six months from months 14-55. At midpoint during enrollment (Month 23), we will conduct a qualitative study on multi-level factors for Aim 4.

2.2 Statistical Procedures: The analytic approach is built upon the investigative team's prior HIV prevention RCTs, which involves an intention-to-treat approach, handling missing data using multiple imputation, and conducting sensitivity analyses to quantify robustness of findings based on models and their assumptions. Descriptive statistics will characterize the sample and measurement distributions to ensure proper application of multivariate methods. We will use multiple imputation to handle missing data. Attrition analyses will identify variables to be

included in MI models to handle nonignorable nonresponse. Preliminary analyses include bivariate analyses to identify unadjusted associations among variables. Variables significantly associated with outcome variables and attrition, as well as significantly different across study arms, will be included as covariates during hypothesis testing. Generalized Linear Mixed Models (GLMM) and Generalized Estimating Equations (GEE) twill be used to account for the nonindependence in measures arising from: (1) “autocorrelation” due to repeated measures with the same person; and (2) “intraclass correlation [ICC]” arising from group membership. The data analytic approach for testing hypotheses with outcome specific models for primary outcomes is presented in Table 4. Analytic strategies for secondary outcomes will follow a similar approach.

Hypothesis testing will implement an intention-to-treat approach. We will employ Generalized Linear Mixed Models (GLMM) and Generalized Estimating Equations (GEE) to account for the non-independence in measures arising from: (1) "autocorrelation" due to repeated measures with the same person; and (2) "intraclass correlation [ICC]" arising from partners who are reporting on conjoint behaviors. Hypothesis testing for Aim 1 follows the form “Assignment to E-WORTH will be associated with significantly lower|higher outcome variable Y at the 12-month follow-up compared to assignment to Streamlined HIV Testing” where ‘lower’ or ‘higher’ is chosen in the direction of reduced risk. The selection of outcome models build upon g (E [(Y_ijx_ij)])=x'_ij where Y_ij is the outcome of interest for the ith participant at the jth time point. The multilevel nature of the data is accounted for by modeling each predictor variable’s coefficient in the vector as dependent on group membership: where the ith participant is a member of the gth group that determines the group characteristics (i.e., elements of the vector w) used to estimate model fit parameters in vector. The components of will be treatment assignment, baseline attribute for the outcome variable, and those variables selected during preliminary analyses noted earlier. The g () function represents a link function that can model the outcome as a function of measurement unit or distribution.

2.3 Power Analyses: From ICC estimates based on the previous WORTH trial, variance inflation factors were calculated to generate effective sample sizes used in G*Power for Aim 1. For cumulative STI incidence, we assumed a 50% reduction (on a background cumulative incidence of 20.5%) to be clinically meaningful to detect via Poisson regression; for behavioral outcomes, projected means, standard deviations, allow for effect size f2 to be calculated and used in G*Power. Results for primary outcomes (Figure 3) indicate that 80% power is achieved for the rate-limiting outcome (i.e., cumulative STI incidence) with an effective sample size of 372 participants; translating to a sample size of 378 participants after accounting for ICC. We then increased the starting sample size to 420 to safeguard against attrition (modeled as a complete loss of 10% of data as conservative estimate since the efficacy trial of WORTH achieved a 91% retention rate at 12-month follow-up and many of those cases provided data at earlier assessment points, thus were not “fully” missing). For secondary outcomes, power analyses participants/arm indicate 80% power to detect differences for effect sizes f2 = .03 to .04 (for = .1 TO .5) with sample sizes as low as 369.

3. Study Procedures

3.1. The Study Team

This research will be undertaken by the Social Intervention Group (SIG) at the Columbia University School of Social Work. SIG is a multidisciplinary research center with a strong infrastructure and experienced researchers and staff. This study benefits from SIG's extensive

clinical trials experience, an investigative team with longstanding collaborative relationships, and the availability of established and tested protocols from previous randomized controlled trials conducted in community correction sites. The investigative team successfully completed an efficacy trial of Multimedia E-WORTH intervention with drug-involved women under community supervision with the New York City Department of Probation. The investigative team will replicate many of the approaches, procedures, and protocols that achieved successful during the pilot trial, which also did not have any adverse events. The study research team will include a project director, a data manager, a clinical supervisor who is a graduate research assistant and 2 research assistants who will be responsible for recruitment, administering assessments and retention of participants. For this study, we will have a service agreement contract with Fortune Society who will hire 6 providers (or 1 full-time equivalent) to conduct the interventions for both study arms. They will not be involved in obtaining informed consent or collecting any data for the study from participants. Fortune Society has multiple longstanding service contracts with the New York City Department of Probation. We will recruit participants from probation, parole or designated ATI program sites located throughout in New York City. We have successfully collaborated with the New York City Department on three randomized controlled trials since 2009. Potential participants in ATI programs or parole will NOT include individuals who are detained in a residential facility or treatment center as a form of sentencing or alternative to incarceration.

3.2. Overview of Study Participation

This randomized controlled trial will be conducted with 420 drug-involved Black women from probation, parole or designated ATI program sites located throughout communities in NYC heavily affected by HIV and STIs. Eligible women will be randomly assigned to: (1) E-WORTH consisting of an individual evidence-based Streamlined HIV Testing session followed by a 4-session group-based multimedia HIV intervention (E-WORTH) or (2) an individual Streamlined HIV Testing session alone (Streamlined HI Testing), which will serve as the comparison condition. Both conditions will be delivered at two Fortune Society Sites (located in Harlem and in Long Island City). If participants live too far to attend sessions at these sites, Fortune staff may also deliver intervention sessions at our community research site or at a NYC DOP site approved by the appropriate DOP Assistant Commissioner. Repeated assessments will occur at baseline and 3, 6, and 12-months post intervention. For participants residing out of New York City during their follow up period, or are otherwise prevented from traveling to study sites to complete a follow up assessment, we will send a link to follow up assessments directly to the participant's e-mail on a password protected PDF document. We will conduct repeated assessments and qualitative in-depth interviews on multi- level factors that may influence the effectiveness of E-WORTH and Streamlined HIV Testing on study outcomes with Fortune providers delivering the interventions, frontline probation, parole or designated ATI program staff (e.g. probation officers), probation administrators and probation clients. Self-reported data on behavioral outcomes will be collected from participants via audio computer-assisted self- interview (ACASI). Primary outcomes will be measured via biological assay for infection by Neisseria gonorrhea, trichomonas, and Chlamydia trachomatis, the most common STIs found among women in NYC.

3.3. Recruitment and Screening of Participants

Recruitment of participants for RCT for Aim 1: Recruitment will follow procedures that have been approved by Columbia University for recruiting participants from probation sites Projects PACT (AAAK0206), WORTH (AAAD5608) and WINGS (AAAK2851). These

procedures have been refined over the course of the investigative team's multiple federally-funded studies that have successfully screened over 1000 potential participants and met target enrollment numbers of participants into HIV prevention trials. We will use various strategies for recruitment, including onsite recruitment at probation, parole or designated/selected ATI program sites, field referrals and by word-of-mouth described in detail under F Section.

Screening of participants for RCT: If a woman expresses interest in being screened for the study, the RA will describe the study and answer any questions. All potential participants will be apprised that their involvement in the study is entirely voluntary and that it doesn't fulfill any court mandate. If the woman indicates that she is willing to be screened, the RA will obtain written informed consent to be screened by reading the screening consent form to the participant and asking if she has any questions about the screening interview before obtaining her signature on the form. After obtaining informed consent, the RA will conduct a 10-15-minute screening interview to determine eligibility and willingness to participate. The screening interview will contain eligibility related items, socio-demographics, and other items that will camouflage the eligibility criteria. Interested potential participants will undergo a written informed consent and complete a 10-15-minute screening in a private room, which will consist of questions about sexual behaviors, history of drug use, and demographic information (see screening form). If a private room is not available, or if the participant wishes to be screened at a later time, the participant will be screened via phone. Once the potential participant's eligibility has been established, the RA will schedule her for an appointment to complete the baseline assessment.

The RA will ask participants who screened eligible to complete a contact information sheet that asks for their address, telephone numbers where they can be reached, and email information using a Contact information sheet. More detailed contact information (Locator Form) is obtained when participants come for the baseline assessment. In addition, participants will be asked to provide the number of children they care for directly using the Child Information Form in the event that childcare becomes a barrier to participation. Potential participants will receive a handout that introduces the study, explains compensation, and contains a contact telephone number. To ensure participants are aware of meeting times, we will send letters to remind them of their baseline assessment, HIV testing and E-WORTH session appointment times. When potential participants are not eligible, we will send a re-screen letter after 90 days to see if they are interested in screening for the study again. At the time of the screening interview, participants will also be asked to sign a HIPAA form.

Participants will be compensated $5 for completing the screening interview. Women who meet eligibility criteria (described below) will be asked to participate in the study, and will be told that their decision whether or not to participate in the study will not affect their status with court, NYC Departments of Probation and Parole, and designated ATI sites, or the criminal justice system. Interested participants will undergo an additional informed consent process, and will be scheduled to complete a pre-intervention (or baseline) assessment at the SIG Community Research Site. During the baseline interview, participants will be asked to provide more detailed contact information using the E-WORTH Locator Interview form. In addition, since the intervention will be delivered electronically, participants will also be asked to fill out an E-WORTH Computer Literacy Questionnaire during the baseline interview to gauge levels of computer literacy. Research Assistants will complete a study check list each time participants are assessed to ensure that all items on the check list are completed.

Recruitment of probation, parole or ATI program staff and Fortune providers for survey (Aim 4)– Prior to the beginning of the study, we will present the purpose and procedures of this study to all probation, parole or ATI program staff at the DOP study sites and to Fortune staff. After our presentation we will ask staff to participate in the survey. Staff, who are interested in participating in the study, will complete written informed consent administered by a research assistant prior to enrolling in the study. During informed consent, potential participants will be apprised that their participation in our study will not affect their position at DOP or Fortune in any way and they may withdraw from the study at any time without questions asked. For the mixed study for Aim 4, we aim to enroll 25 staff probation, parole or ATI program officers and 10 administrators (supervisory Probation, parole or ATI program Officers/Branch Chiefs/Other Managers) and the providers from Fortune Society (1 per site, n=5) delivering E-WORTH and Streamlined HIV Testing condition will be asked to complete ACASI structured survey the month prior to starting the RCT with repeated assessments every 6 months through month 45.

Recruitment of participants and probation, parole or ATI program staff for in-depth interviews (Aim 4) — From all the NYC probation, parole or ATI program sites, the Project Director will randomly select 4 women on probation, parole or ATI program who enrolled in the study (a total of 20 participants) and ask them to complete the in-depth survey. The Project Director will also randomly select 10 probation, parole or ATI program officers and 10 Probation, parole or ATI program Administrators from those who completed the ACASI structured survey and invite them to participate in an in-depth interview. In addition, the Project Director will invite all service providers from Fortune society who delivered both intervention conditions (5 service providers) to participate in an in-depth interview. The project director or an RA will contact all probation, parole or ATI program staff and study participants who have been selected to participate in the in-depth interviews by phone or mail. The project director or an RA will describe the purpose of the in-depth interview and answer any questions that randomly selected staff and study participants may have about the in-depth interviews. If staff and study participants indicate that they are willing to participate in the in-depth interview, the project director or an RA will administer informed consent.

3.4. Assessments:

Participant Assessment for Aim 1: When a participant arrives for their pre-intervention assessment, they will undergo a shortened version, 5 minutes, of the screener to confirm eligibility. If a woman no longer meets eligibility criteria, she will be compensated $10 and will no longer participate in the study. Women who meet eligibility criteria will undergo a pre-intervention assessment. This assessment will last approximately 60 minutes, consisting of demographic information, history of criminal justice involvement, drug and alcohol use and dependence, sexual and drug-related HIV behaviors, HIV treatment and care, intimate partner violence, depression and PTSD, service utilization, and social support. RAs will schedule repeated assessments with participants that will take place at baseline and at 3, 6, and 12 months post- intervention. For all assessments, participants will complete the ACASI surveys on computers with headphones at a computer work station designed for privacy so that others cannot see their responses on the computer. Participants will be given the option to complete the baseline survey at SIG's community research office at 3280 Broadway or on site at Probation, parole or designated ATI program or Fortune Society sites.

Process data collected for Aim 4: Providers will collect attendance data for each intervention session using the E-WORTH attendance form and provide information on number and general

type of referrals to services using participant ID numbers. Providers will also be asked to complete a session adherence form for each session that assesses the extent to which core elements and activities of the session were covered and the quality of delivery of each activity using a rating scale. At the end of session 5, E-WORTH participants will be asked to complete an anonymous ACASI client evaluation of intervention that assesses their level of satisfaction with the intervention and provide. Streamlined HIV testing alone participants will also be asked by their providers to complete an anonymous self-administered written evaluation of the session. In addition, the computerized multimedia E-WORTH tool will generate data on whether or not participants completed each session activity and the amount of time spent on each activity. Staff survey Assessment for Aim 4: For Aim 4, probation, parole or ATI program officers (n=25), administrators (n=10) and the probation, parole or ATI program providers from Fortune Society (n=10) delivering E-WORTH and Streamlined HIV Testing will be asked to complete an ACASI survey that will take approximately 45 minutes at month 9 with repeated assessments every 6 months through month 45. After completing informed consent, probation, parole or ATI program staff and provider participants will be asked to complete an ACASI survey which they may complete on line using the secure Dat Stat Server described below. This survey will include questions and scales that assess multi-level factors that may influence the fidelity of implementation and effectiveness of both intervention conditions on study outcomes, including: socio-demographics and professional training of staff and providers, their attitudes toward the E-WORTH and streamlined HIV testing condition, provider self- efficacy in delivering interventions, Organizational Readiness to Change, and Organizational Climate.

In-depth interviews of study participants, Fortune providers and staff for Aim 4: To help us open the “black box” of the delivery of E-WORTH and Streamlined HIV Testing intervention in probation, parole or ATI program sites, we will collect qualitative data to identify multi-level factors and processes that enhance or diminish the fidelity of implementation, which would otherwise be unobserved in the quantitative analysis. At the midpoint of trial (Month 23), we will conduct in-depth interviews with 10 Fortune providers who delivered the sessions, 20 probation, parole or ATI program officers, 10 Probation, parole or ATI program administrators, and 10 E-WORTH and 10 HIV testing information participants randomly selected from each site. All interviews will be audio-recorded and transcribed. The project director or GRA for the study will be trained to conduct in-depth interviews with selected Probation, parole or ATI program staff and study participants and Fortune providers who deliver the interventions for both conditions.

The first section of the interview will be administered to all Fortune providers delivering the interventions, probation, parole or ATI program officers and administrative staff to identify multilevel factors or processes that may influence the fidelity of implementation of the interventions, such as: perceptions about the benefits/disadvantages of both interventions for their site, their perceptions of clients’ reactions to both interventions, their perceptions about their probation, parole or designated ATI programs organizational climate, and their attitudes towards criminal justice. The second section will be delivered only to Fortune providers delivering both interventions, which will explore their perceptions about: what core components of E-WORTH are relevant/useful, difficulties they experience in delivering intervention activities, how participants responded to the cultural tailored content of the E-WORTH sessions, group dynamics, barriers to engagement of participants and their perceptions of what training, technical assistance and supervision is needed to deliver both interventions and any unanticipated issues or incidents that they experienced. In-depth interviews with 20 randomly selected participants will explore their attitudes towards

probation, parole or ATI program staff and Fortune providers and any barriers that may have impeded their participation in the intervention.

All interviews will be audio-recorded and transcribed. We will construct a qualitative dataset that will include identification of each transcript, inclusion of relevant demographic data by provider/probation, parole or ATI program staff and by probation, parole or designated ATI program sites that will aid in data analysis of key themes that emerge. A preliminary set of analytic coding categories (closed codes) will be assembled based on concepts, ideas, themes and patterns that characterize multi-level theory- driven factors or processes described above. This set of codes will be continually updated through a process of contrast and comparison. The initial or open-coded data is then organized under the analytic category list. Qualitative data will be analyzed using the Atlas 5.2 software system. Key themes and patterns related to how multi-level moderators may influence implementation of the intervention from the qualitative findings will be compared and contrasted to the quantitative study results for specific multi-level theory-driven moderators for Aim 3 described below and process measure data on fidelity of implementation. The qualitative data will be used to interpret the outcomes of the study.

Usage data collected from multimedia website: The web program for E-WORTH will collect log in and log out date and time. Any time a resource such as a video or document is accessed, the web program will track the time and date of access, and for videos, the duration and proportion of resource viewed. Participants will be asked to respond to questions that assess from which type of location they are accessing the web program from and with whom they are viewing the website with.

3.5. Biological Assays

Collection and Testing of Biospecimens for STIs: To complement self-reports and provide a more objective outcome, we use biological assays for gonorrhea, Chlamydia, and trichomonas. These STIs are selected based on their high prevalence and incidence in this population, the availability of accurate tests, and because each can be definitively treated with a single oral dose of antimicrobial medication. We will assess STIs with DNA amplification assays on self-collected vaginal swab specimens (also referred to as STI testing) from the women, which have several advantages including stability for transport and ease of collection. Immediately following the baseline assessment, the clinical research coordinator (CRC) will provide pre-test counseling on STIs to the participant and then ask her to collect a vaginal swab (self-administered by the participant) that will be used to test for gonorrhea, Chlamydia and trichomoniasis. The clinical research coordinator will then provide follow up post-test counseling to notify participants of their results and treatment referrals as needed. Each participant will be asked to obtain one vaginal specimen by inserting a sterile Dacron-tipped swab about 2.5 inches or as far as comfortable into the vagina, rotating it for 15 to 30 seconds, and removing it. The vaginal swabs are placed into separate specimen transport packaging. Refrigerators will be utilized for storing specimens between shipment dates. Specimens will be shipped within 72 hours of collection. All specimens will be shipped and processed by Bioreference Laboratory in New York City using standard handling procedures in specially designed biohazard containers suitable for Dangerous Goods Shipping according to International Air Transport Association (IATA) guidelines. Bioreference has worked on other currently NIH funded studies involving STI testing, including another randomized controlled trial with probationers (Project PACT). We will not conduct biotesting or store biospecimens at DOP sites. Biotesting will be conducted at Fortune locations or at SIG's community research office.

Notification to Participants of STI Results: For delivery of STI results, participants are given a card with a unique study ID number, the name and contact numbers of the CRC and Project Director, and the expected date to contact the CRC for test results. Participants, who test positive for any of the STIs, will be informed privately by the CRC, counseled, and referred for treatment (see treatment protocol below). While all attempts will be made to deliver STI results in person, the CRC will provide STI results over the phone to participants who indicate they are not able to report to the study site in person in an effort to expedite treatment. The research assistant will obtain participant consent to deliver STI results over the phone at baseline. Detected STIs are treated according to CDC guidelines. After notification, the participant will be directed to seek services at her preferred healthcare provider. If participants do not have a health care provider, they will be referred to free STI clinics as well as a healthcare provider that is within a 5-minute walk, has a sliding scale and accepts Medicaid, and has worked with SIG in prior and ongoing HIV/ STI clinical trials.

Biospecimen Testing Procedures for Streamline HIV testing and counseling: Immediately following randomization participants in both conditions will receive HIV testing as part of the Streamline HIV testing intervention and again at the 12-month follow-up assessment. Fortune providers who will be delivering the intervention condition or the CRC at the 12-month follow up will ask all participants to provide an oral specimen, using the OraQuick test procedures to confirm their HIV serostatus. Sensitivity and specificity for the OraQuick oral fluid assay are 99.3% and 99.8% respectively. Results are available from the rapid test in 15 minutes. If the first OraQuick ADVANCE assay is nonreactive, then no further testing will be conducted. If the rapid test is reactive, then the participant will be informed about the results and a confirmatory OraQuick test will be conducted immediately. Regardless of whether the confirmatory rapid test is reactive or non-reactive, the participant will be informed of the confirmatory results and referred for further confirmatory HIV testing and, if needed, treatment and care. All participants will receive standard post-test HIV counseling from the Provider or the Clinical Research Coordinator, who will be certified to conduct such counseling. Participants who are identified as HIV seropositive will be informed in private and referred to clinical sites for further diagnostic evaluation and care. The collection, results, and referral procedures for HIV and STIs have been used successfully and without adverse events in the investigative team's prior and ongoing federally-funded clinical trials of HIV preventive interventions with at-risk and/or marginalized women. During the informed consent process, we will apprise participants that we will report positive Chlamydia, gonorrhea, or HIV tests to the New York City Department of Health (NYCDOH), and that this is the same procedure followed when people are tested in a private physician’s office. We will explain all procedures in the consent form, up front, including potential risks and benefits (especially the benefit of detecting and getting treatment for an STI and HIV, when symptoms are not observable). We have not found this to jeopardize trust in any way. In fact, the openness of the consent form may enhance trust.

Legal HIV/STI Reporting and Tracing Requirements: If a participant tests positive for gonorrhea, Chlamydia, or HIV, the CRC will complete a Confidential Morbidity Report. The “Confidential Morbidity Report,” which identifies the STI-positive individual and the specific STI diagnosis, must be received by the NYCDOH within two weeks of the CRC receiving the participant's STI results. Upon the receipt of the Confidential Morbidity Report, NYCDOH will initiate contact tracing as per standard procedures. Upon referral, NYCDOH protocol requires that diagnosed individuals be interviewed by a trained Disease Investigation Specialist and asked about their

sexual contacts for contact tracing. NYCDOH will ask infected individuals whether they would like to disclose positive tests to their partner(s) and bring the partner in for treatment, or whether they would prefer that DOH staff contact the partner. Individuals testing positive for STIs, including HIV, however, are not mandated to, nor can they be coerced to, disclose the names of their sexual partners. Both scenarios offer opportunities for the participant to choose whether or not to disclose with or without support.

Protocol for STI Treatment: All participants who test positive for gonorrhea, Chlamydia and trichomoniasis will be referred for treatment with a single dose antibiotic therapy that will be administered by licensed physicians who are participating providers for SIG's current studies, or if the participant prefers, by her primary care provider. All SIG providers accept Medicaid and all other types of insurance. All SIG providers have received human subjects training. If a participant is uninsured and can't afford treatment, SIG will pay for treatment out of in-kind funds. We anticipate that very few participants who test positive for an STI will not have Medicaid or other insurance based on our current studies with similar populations. The participants' STI and HIV serostatus will be kept strictly confidential and known only to the participant, the CRC, and the physician who is treating them. These procedures are being used in our current studies and are in compliance with the most up-to-date New York State and New York City laws and standards. Of critical importance is the confirmation of participant's treatment. From both ethical and methodological reasons, it is imperative that treatment be completed promptly. Thus, the CRC will work closely with the participant to confirm that STI treatment has been received. After notification, the participant will be treated immediately or scheduled to return to a designated clinical site for treatment by a project clinician. For both ethical and methodological reasons, it is imperative that treatment be completed promptly and confirmation of intervention is obtained. Participants are treated with directly observable state-of-the-art single-dose oral therapy to minimize potential non-adherence to multi-dose medication regimens. We have been successfully using this STI treatment protocol in Project PACT and Project WORTH.

Protocol for linking participants who test positive for HIV to treatment: Receiving a positive test result for HIV may produce psychological stress. The study providers and CRCs will be trained to provide post-test counseling, identify and address any psychological distress associated with receiving a positive HIV result and provide enhanced referrals for HIV care. We will follow current CDC recommended guidelines and evidence-based strategies for ensuring linkage to HIV treatment and care. Specifically, our staff will be trained on the evidence-based ARTAS (Antiretroviral Treatment Access Service) intervention that was shown to effectively link persons newly diagnosed with HIV to primary medical care). Our co-investigator Dr. Lisa Metsch was one of the developers of this interventions and has provided trainings throughout the U.S on this intervention. The Centers for Disease Control and New York City Department of Health currently recommends ARTAS as an active linkage intervention. Participants, who [newly] test HIV positive during the streamlined HIV testing intervention session or at the 12-month follow up assessment, will be offered information about local resources and referred to clinical sites for further diagnostic evaluation and care. Providers or the CRC will help participants identify which HIV care clinical site will work best for them and offer to call the clinic to make an appointment. Providers will also give newly diagnosed participants their contact information and encourage them to call if they have any follow up questions or need additional assistance to access HIV care. Although we offer this HIV testing at no cost to the

participant, she may choose not to get tested and still be able to participate in the study (i.e. willingness to take the HIV test is not an eligibility criterion).

3.5. Randomization
Consistent with an intention-to-treat approach for data analyses, participants will only be considered enrolled in the trial when they have been randomly assigned to a study arm. We will implement urn randomization to “force” a balance (without adding bias) between study arms with respect to the number of women per arm so that half of the women will be randomized to E-WORTH and immediately receive the individual Streamlined HIV test session followed by 4 E-WORTH group sessions and half will be randomized to and immediately receive the Streamlined HIV Testing session alone. The randomization program, which has been adapted from our previous RCTs, will also feature a combination of randomization and assignments to balance the number of women per condition per site so that the number of women assigned to each condition will be distributed across probation, parole or designated ATI program and study sites. In addition, participants randomized to the intervention arm will complete the intervention individually if less than 4 participants are enrolled at a given site in 28 calendar days. Additionally, the assignment window during the Treatment as Usual condition assignment phase will open after the first participant is assigned. We will explain the randomization process to participants during informed consent. The randomization and assignment protocol will also ensure moderators of intervention effects assigned to study arms, thus minimizing the possible introduction of bias that can occur with urn randomization or block permutation techniques.

3.6. Intervention Conditions
Streamlined HIV Testing Alone Condition: The streamlined HIV testing with information Alone condition was tested and found to be effective in two recent large scale multi-site studies that were conducted in the NIDA Clinical Trials Network (CTN 0032 and Project AWARE) and led by our co-investigator Dr. Lisa Metsch. This study will employ the Streamlined HIV Testing protocols used for these trials. Fortune Society staff will deliver a 5-minute HIV testing information session to HIV Testing participants in a private space that will describe the rapid testing procedure, timing for and meaning of test results, explain the window period during which an antibody test might be negative. Providers will briefly describe what HIV infection is and give them the pamphlet from the HIV testing kit, which provides an overview of transmission risks and effective strategies for reducing. This streamlined HIV testing intervention is consistent with New York state law that does not require risk reduction counseling at the time of HIV testing.

Participants are then offered a rapid Oraquick HIV test and escorted to a waiting room for 20 minutes until the test results are ready. For participants who test negative, Fortune providers will conduct another 5-minute explanation of the duration of the window period during which the test might be falsely negative. HIV post-test counseling for participants who test positive for HIV will follow CDC-recommended ARTAS protocol that will include providing psychological support and referral for confirmatory HIV testing and care, identify and address barriers to care, and review plan to reduce risks and discuss disclosure issues. WORTH Intervention Condition: WORTH consists of one Streamlined HIV Testing session described below followed by four 1.5-hour group sessions of 3-8 women that will be delivered by a Fortune Society facilitator provider at the Fortune Society sites. The main multimedia components used in the WORTH: (1) Narrativity: Use of videos with fictional characters who resemble different life stories of Black women affected by HIV/AIDS, drug use and criminal justice. These characters provide inspirational messages, model group support and core risk reduction skills; (2) Skill acquisition using

simulated video vignettes that provides instruction and demonstration of core skills (e.g., safer sex negotiation and problem-solving skills, technical condom use skills) using culturally congruent role models followed by simulated video vignettes of role play scenarios where participants are prompted to identify common pitfalls in using skills and to rehearse appropriate responses; (3) Individual interactive exercises and logs that are designed to enhance participant's recall of core knowledge and to provide a confidential space for participants to track their individual progress in reducing risky behaviors and achieving risk reduction goals, current and ideal future social networks, and the types of support for sustaining safer behaviors, and future plans; and (4) Facilitator interactive guide which acts as a road map, prompting the facilitator to move sequentially through each activity without the need to rely on notes or memory. The basic format of each E-WORTH session remains consistent following a sequence of 5 steps: (1) an opening (quote, song, poem) which will provide a brief culturally relevant point of inspiration to engage participants; (2) Check-in to review material from the previous session, (3) a discussion to raise awareness of links between IPV, drug use, criminal justice involvement and HIV risks; (4) a skills-building component relevant to the discussion; and (5) an individual computerized rating of their sexual and drug risks; (6) an individual computerized to set appropriate risk reduction goals and plan of action which they can text to themselves. (7) identification of service needs and appropriate referrals using a computerized service tool. E-WORTH more exclusively tailored to the realities of Black women on probation, parole or ATI programs, including: (1) adding recent HIV/STI statistics for Black women in NYC on probation, parole or an ATI program to raise awareness about the epidemic in their communities; (2) using only Black characters and narratives, incorporating Afro-centric messages that highlight historic resiliency of African-descended women to enhance protective practices and focus on the unique challenges (e.g., stigma, racism, discrimination) Black drug-involved women on probation, parole or ATI encounter; and 3) upgrade the web-based platform so that it may be used with tablet and mobile phones. See Attached E-WORTH intervention manual with login information to the computerized E-WORTH intervention.

3.7. Compensation

All participants will be reimbursed up to $340 for their time for completing the screening and the assessments. Below is the compensation that participants or potential participants in the randomized controlled trial study will receive for study activities. This includes incentives to cover travel expenses to office sites.

$5 for eligibility screening interview
Two-fare metrocard currently valued at $6
$55 for enrollment (that is $35 for completing the baseline assessment, vaginal swab specimen - also referred to as STI testing, and $20 for orientation and HIV testing)
$20 bio retesting
$50 for getting Bio Results and Bio Treatment in the same day. Transportation and change of contact info is included.
Plus, an extra $5 if the participant arrives on time for the enrollment meeting
$100 ($15.00 for each of the 4 intervention sesessions you complete + an extra $5 for being on-time to each session. You will also receive $6 for transportation.)
$50.00 for completing the 3-month follow-up interview
$60.00 for competing the 6-month follow-up interview
$65.00 for completing the 12-month follow-up interview and STI test and HIV test
$50 CBO referral bonus (10 participants)
$20 participant referral bonus (10 participants)
$-15 childcare
$5 change of contact information

$35 Graduated participants will be invited to have a qualitative interview

They will also receive a light meal at each visit to the office.

In addition, participants and CBO staff are also eligible to earn the following if they refer others to the study:
$5.00 for every person they refer to the study that is later screened. When a CBO staff successfully refers 10 eligible participants who enroll and attend session one, they will receive a bonus of $50. When a participant makes 10 succesful referrals, they will receive a bonus of $20. The person referred must meet the basic criteria of the study. That is, she must be Black, 18 years or older and must be on probation or recently completed probation in the past 90 days.
$10.00 for every person referred to the study who is later enrolled and completes their first follow up meeting.
$15 Childcare incentive will now be offered to women for assessments.
$50 incentive now will be offered to be able to encourage hard to reach participants who were also tested positive for an STI and need to be treated as soon as possible before they are due for 12 month follow up appointment. Hard to reach participant will be provided her STI results and escorted by a Research Asisitant to the most convenient Free STI Clinic. Incentive is included delivering the STI Results, Getting Treated and Confirming the Treatment, Transportation and Changing Contact Information when its all done in the same day

Lastly, participants are also eligible to receive an additional $5.00 if they change their contact information (i.e phone number, mailing address, etc…) and report the change to a member of the study.

For the mixed methods study with Fortune Society employees who are assigned to the E-WORTH Project will receive the following compensation for study activities. NYC Department of Probation does not allow their staff to receive any compensation for study activities.

$30 for survey interviews (repeated every six months from the study start to month 56)
$30 for qualitative interview. ($30 + $5 for transportation= $35)

3.8. Retention
This study will implement tracking procedures used in our previous research that have proven effective in achieving high retention rates of low income, drug-involved and criminal justice-involved populations. These procedures include: (1) administering a detailed locator package at baseline; (2) assigning cases to RAs to track from baseline to the 3-month follow-up; (3) verifying locator data and updating data at every assessment; (4) maintaining biweekly phone, text, email, or mail contacts with participants during the follow-up period (i.e., birthday cards, holiday cards, appointment notices, thank you cards, check-in calls); (5) implementing returned mail procedures; (6) conducting outreach to unconfirmed cases; and (7) making reminder calls to participants one week prior to their appointment and 24 hours prior to their appointment. In collecting locator information on alternative contacts (i.e., friends, neighbors, family members, and service providers), RAs will apprise participants that they will not disclose their study participation to the contacts, but simply introduce themselves as Columbia University staff and leave a message with the alternative contact for the participant to call them back. RAs will be trained to engage participants in identifying and overcoming barriers to attendance at follow-up sessions. The participants will receive monetary compensation for assessment sessions (see above compensation section or specific details).

3.9. Quality Assurance

All sessions for both conditions will be digitally recorded with 10% randomly selected for QA to rate how closely facilitators adhered to the intervention protocol and the overall clinical quality of delivery each session activity. QA data are reviewed, processed, and addressed/troubleshot during regular Facilitator supervision. If QA data for a Facilitator is found to fall in the insufficient range after attempting to rectify the problem, the Facilitator will not be assigned new couples (but will continue with existing couples to ensure continuity for participants with all recordings reviewed) until the Facilitator has successfully become "recertified" via re-training (e.g., additional readings, mock role-plays). Any detected instance(s) of contamination by facilitators or improperly handled responses to participants bringing up information from the other intervention will trigger immediate corrective action

4.0. Selection and Training of Staff

All study personnel will complete a common training that includes the impetus, goals, organization, protection of human subjects, and rules/regulations. Research Assistants will receive additional hours of structured and standardized training for specialized tasks. Facilitators from Fortune Society will be trained to deliver both intervention arms. The facilitator training course (one for each condition held at different times) lasts 5 days and modeled on similar trainings conducted during the investigative team's prior and ongoing couples-based HIV prevention RCTs. All study staff will complete all required human subjects trainings designed to ensure the protection of the rights and ethical treatment of human subjects. All personnel involved with or supervising the implementation of biological assays will also complete the blood-borne pathogen/infection control laboratory training. Documentation of sufficient and appropriate human subjects and laboratory training will be achieved by submitting a modification to the IRB protocol with the newly hired and trained staff.

4. Study Drugs or Devices

N/A

5. Study Questionnaires

The following questionnaires are attached to the IRB protocol:

Screening Questionnaire: The eligibility questionnaire will be administered in a face-to-face interview by a trained RA. The screening interview will take approximately 15 minutes. Before completing the baseline, an RA will administer a face-to-face interview with selected eligibility items from the Questionnaire to confirm eligibility of participants.

Baseline/Follow up assessment: This assessment will be used for baseline as well as 3-, 6- and 12-month follow up assessments.

- Immediate post-intervention evaluation questionnaire: This evaluation questionnaire will be administered individually at the end of the final session for E-WORTH participants and at the end of the streamlined HIV testing alone session for participants assigned to this condition. There is an evaluation form specific for both intervention arms.

6. Study Subjects

We plan to enroll 420 women in each of three intervention conditions, for a total of 420 women. In order to obtain the desired number of participants meeting eligibility criteria, we estimate screening up to 1680 potential participants.
Inclusion criteria for women in this pilot study are as follows:
(i1) She is 18 or older.
(i2) She is currently supervised by a criminal justice entity, such as a community court or probation, parole or ATI.
(i3) She reports engaging in unprotected vaginal or anal sex with a male partner in the past 90 days
(i4) She reports any illicit drug use or binge drinking or in alcohol or drug treatment in the past 6 months.
In addition, potential participants will be ineligible if any of the following criteria are met:
(e1) Ability to speak and understand English is not sufficient to participate in assessments or intervention sessions.
(e2) The woman's sexual activity is limited to a monogamous relationship lasting more than 12 months, and she has not engaged in any of the additional HIV risk behaviors in the past 90 days:
-Having sex with more than one partner
-Having sex with a partner known or suspected to be HIV positive or an injection drug user (IDU)
-Sharing injection drug use needles or equipment
(e3) The woman is actively trying to get pregnant/have a baby.
(e4) Inability to complete informed consent process due to a psychiatric or cognitive impairment.
(e5) The participant was born male.
The research staff who is administering the consent for screening and intervention will determine capacity to consent by asking comprehension questions such as "what are you agreeing to do by consenting to this research study?" and "how long will the screening interview take?" Additionally, research staff conducting will be trained to assess primary mental status, including orientation to person, place, and time, and signs of major cognitive impairment. In the end, if the research staff is not completely convinced that the participant can give full informed consent, the participant will be excluded from the study.

7. Recruitment

Active recruitment will occur on site at DOP probation offices and at parole or designated ATI program sites in NYC that are selected by DOP's Deputy Commissioner for Adult Operations or his or her designee and the leadership of parole or designated ATI programs located in NYC. The Project Director will communicate as requested with DOP, parole or ATI program leadership personnel regarding recruitment strategies and will present a draft recruitment schedule to DOP's Deputy Commissioner for Adult Operations or his or her designee, and representatives from parole or ATI programs in NYC for their review. The Project Coordinator will provide an invitation letter to DOP's that contains the basic eligibility criteria and contact information of the study, to be send to their clients who might be a potential participant. Columbia research assistants, who have been certified in human subjects and HIPAA training, will recruit participants by approaching female clients after completing probation check in at kiosk stations, intake sessions or probation appointments and at parole or ATI program sites, and ask permission to talk to them about E-WORTH. We will also present the study to Probation, parole or ATI program staff.

If clients express interest, the research assistants will give them a project information flyer that provides a telephone number and basic information regarding the study. These flyers and other promotional materials must be reviewed and approved by DOP's Deputy Commissioner for Adult Operations or his or her designee.

Recruitment will also occur via radio broadcasts on radio stations (David Rothernberg's show on WBAI – Mr. Rothernberg's is the founder of the Fortune Society, Unlocked with Ricky Jones on WHCR 90.3 FM – radio program targeting individuals involved in the criminal justice system) to which target population commonly listen.

RAs will introduce potential participants to the general nature of the study and other preliminary logistics (e.g., time, compensation). Clients will be informed that participation in the study is completely VOLUNTARY. If a participant chooses to participate or chooses not to participate, or chooses to participate and then decides not to participate, they will be advised that the study activities would not affect their status with the court, probation, parole or ATI programs or criminal justice system.

Passive recruitment will involve flyers and advertisements that may be posted or electronically delivered in locations in probation, parole or ATI program sites and community-based organizations that provide services to women on probation, parole or an ATI program.

In addition, the Project will play a video at Department of Probation, parole or ATI programs sites for the sole purposes of recruiting new participants in the study. The video will be played on DOP, parole or ATI program screens in areas in which women wait to be seen by probation, parole or an ATI program personnel.

In addition, study participants or women from probation, parole or an ATI program who screen ineligible will be asked to refer other potentially eligible women on probation, parole or an ATI program without revealing the exact eligibility criteria. Screened participants will also be asked to refer individuals to the study using a coupon referral system. We have successfully used these recruitment strategies in our past and current RCTs that we have conducted in probation.

Research staff will then ask the woman to meet in a private room to discuss screening procedures. This private room is in an area away from probation, parole or an ATI program staff and other clients. If the woman participant is not comfortable completing a face-to-face screening at the probation, parole or an ATI program office, research staff will arrange one of two alternate procedures: 1-the participant will be scheduled with a face-to-face screening interview at the Social Intervention Group research site, or 2-the participant will be instructed to call the study 1-800 number when she is in a private setting in order to be screened by phone. At the conclusion of screening, if the female on probation, parole or an ATI program meets eligibility criteria for the study and is willing to participate, a baseline assessment will be scheduled at the Probation, Parole or an ATI Program Site or the SIG Community Research Site. When a participant attends the baseline assessment, she will first be administered a brief confirmatory screener to re- establish major eligibility criteria (if the baseline occurs more than 24-hour after the initial screening). If the participant no longer meets eligibility criteria, she will be given $10 in compensation and will no longer participate in the study.

8. Informed Consent Process

8.1 Informed consent for participants for mainphase trial: If the participant still meets eligibility criteria, research staff will again describe the study, and the participant will be consented for participation in the study. The informed consent process will emphasize that involvement in research will include a baseline assessment, four intervention sessions, and three follow up assessments, for which the participant will be compensated. She will also be reassured that her decision not to participate or continue in the research study will not result in consequences from the court, NYC Department of Probation, Parole, ATI programs or the criminal justice system. If the participant agrees to take part in the study, she will sign the consent form and will be administered the baseline assessment and HIV/STI testing. After the baseline assessment and HIV/STI testing, the participant will be randomized into one of two conditions, and continue through the study as described in study procedures.

Research Assistants will go through the informed consent process with potential participants immediately prior to conducting the initial and the confirmatory screening interviews as well as at the baseline and all follow-up CASI assessments. In addition, Research Assistants will administer the HIPAA consent form at the screening interview.

Obtaining informed consent will be documented with the use of a written consent form. The consent form provides details of the study procedures, risk, benefits, nature of confidentiality, study contact information, the nature of voluntary participation. The consent process also covers the information on reimbursement for time. Data will be destroyed with no questions asked upon a subject's request. The consent form will state that participants may withdraw from the study without penalty at any time for any reason with no explanation. Participants will also be asked about agreeing for the intervention session to be audio recorded solely for the purposes of quality assurance. The consent form indicates that such agreement is not a requirement to participate in the study and informs the participant that any portion or the entire recording will be erased upon her/his request at any time, including during or any time after the interview. Before a participant signs the informed consent form, staff will thoroughly review the form, ask if the participant understands the content of the consent form, and answer any questions that may arise. As noted above, informed consent will not be considered obtained (and thus, the participant excluded from the study) if a potential participant is unable to state the following after reviewing the consent form: (1) the nature and extent of participation in the study; (2) the risks involved with participation; and (3) the potential benefits of participation in the study. Informed consent for Probation, parole or an ATI program client participants for in-depth interviews: We will follow the general same consent procedures described above for participants who are randomly selected to complete a face-to-face in-depth interview. For randomly selected participants who express interest in completing an in-depth interview. Informed consent for Probation, parole or an ATI program staff and Fortune providers for Aim 4 study activities: For probation, parole or an ATI program staff and providers who express interest in being interviewed in the longitudinal survey, research assistants will similarly go through the informed consent process in a private location and ensure participation is entirely voluntary, stressing that participation is in no way required for employment purposes, and their employer will not know whether or not they chose to participate.

9. Confidentiality of Study Data

All data are collected specifically and only for the proposed study. Data management activities and procedures will utilize the electronic data management systems used in all recent and current SIG studies. These protocols were designed to enhance the efficiency, security, and integrity of study data. This system includes: (1) secure and confidential WWW-based (a) scheduling information for RAs , (b) automatically generated summaries to tailor recruitment efforts, (c) uploading of digital audio recordings for QA protocols, and (d) collection, monitoring, and summary reporting of data relevant to day-to-day operations of the study (e.g., petty cash disbursements); and (2) custom-programmed data entry software to ensure consistency, integrity, and security/confidentiality of the transfer of written information into electronic databases. Additional procedures are described below. Data collected using CASI during baseline and follow-up assessments are automatically recorded/stored by the computer (i.e., no hardcopy of the data is generated). The study will use DatStat products and services for creation and administration of CASI as well as management/transfer of CASI data. DatStat data security meets strict government regulatory compliance. DatStat delivers SSL (Secure Socket Layers) and our secure key is provided by VeriSign. As respondents move through the assessment, responses are encrypted for transit between the respondent's browser and the DatStat server using SSL and 128-bit encryption. DatStat is used by researchers across the country and is very familiar with strict research methodology, security needs, and requirements of/responsiveness to human subject protection, IRB, and HIPAA issues.

Digital recordings of in-depth interviews, in-depth interview transcripts and survey data from the CASI interview with Fortune providers will be assigned a study identifier code. Only the PI and the Project Director will have password protected access of the encrypted file linking names of Fortune provider participants and their study identifier code. Participants will be assigned a unique identifier code that is different from their study identifier code to log into the multimedia web program. The file linking the web program identifier and the study identifier will only be accessible to the PI and the Project Director. The web program will only collect usage data regarding the resources in the program. The program will not collect personal identifying information such as email addresses, usernames, IP addresses, or names. Usage data that are collected by the web program will be password protected and accessible only to the PI and the Project Director.

The Project Director will post recruitment/interview schedules for RAs on an encrypted password protected web page dedicated to the study. At the end of each day, RAs complete a daily, on-line summary form which includes data entry fields concerned with operational data (e.g., number of potential participants approached, number and reason(s) for refusal). Although the dedicated web page is password protected, no identifying information is posted/submitted by research staff because web server resources rely on Columbia University technical staff members who are not involved with the study as well as the frequency of security threats discovered by manufacturers of common web browsers (e.g., Microsoft). However, we prefer to use this WWW interface because summary reports (e.g., recruitment rates by RA, by study site, etc.) can and will be automatically generated and e-mailed weekly to the PI and Project Director. These procedures have been implemented without incident by the SIG investigative team in other studies. Study staff will also use the internet to transfer and securely store digital audio recordings used for quality assurance purposes. Digital recordings are transferred and stored on a dedicated FTP server maintained by and accessible only to SIG staff. Centrally storing all recordings on a password- and firewall-protected computer enhances security (e.g., all attempted accesses, whether successful or unsuccessful, are automatically logged and reviewed weekly by a

dedicated SIG computer support staff member) and integrity (e.g., automatic backup of recordings using 128-bit AES encryption onto optical media). These procedures were developed by Dr. Wu and currently in use for all current SIG studies. At the completion of the study, all digital files with identifying information—including audio recordings—will be destroyed by using software that meets DoD 5220.22-M specifications for files on magnetic media, overwriting files stored on the flash memory used by digital recorders, and physically shredded for files stored on CD/DVD media.

Safeguarding the confidentiality of personal data reported in verbal questionnaires or during other data collection/documentation (e.g., HIV test results) is maintained through a tear sheet procedure; the participant's name is removed from the forms after initial checking, leaving only an identification number, which ensures the confidentiality of the data throughout the study. All interview data are coded to preclude participant identification; no personal identifiers as defined by the Health Insurance Protection Accountability Act (HIPAA) will be included in screening or assessment data instruments with participants and Probation, parole or an ATI program staff and Fortune providers. Research staff will verify that all screening instruments are gathered before they leave the data collection site. All data from screenings will be coded and entered into a database. All data files will be encoded using 128-bit AES encryption and password-protected on end computer devices with both hardware and software firewalls. Electronic sensitive study data will also be stored on Columbia University's secure and HIPAA approved Courseworks multi-user system site.

In addition, the informed consent, locator form and any signed paperwork are kept in a separate folder and to not contain the research identification number. A name/code number roster will be kept locked in a file cabinet separate from the assessments. All research forms and instruments are stored in locked file cabinets in a private office in an access-controlled building. Additionally, Dr. Gilbert has secured a Certificate of Confidentiality from the NIH to avoid subpoena regarding confidential information from subjects. Any attempts to subpoena research information will be immediately reported as an adverse event to the principal investigator and the IRB.

10. Privacy Protections

All face-to-face screening interviews and face-to-face in-depth interviews will take place in a private room either at the probation, parole or an ATI program site, a Fortune site or at our community research site.

All ACASI (Audio Computer-Assisted Self Interview) interviews will take place in an enclosed cubby space or kiosk with head phones so that others may not hear questions or view responses. Although some research activities may take place within a criminal justice setting, all information collected in the interview is confidential, and will not be shared with any criminal justice entity. This includes information about illegal activity, such as drug use. Probation, parole or ATI program department personnel have affirmed that they will not seek identifiable information on research participants. The only instance where research staff are required to release identifiable information is in the case of imminent harm to yourself or others, or if there is a report of child abuse or neglect. We have obtained an assurance letter from Fortune Society andMemorandum of Understandings (MOUs) from the NYC Departments of Probation, Parole and designated ATI programs providing assurance that they will not seek the data collected

about subjects. The general letter of introduction will be composed for the intervention team to introduce them to the supervisors of each probation, parole or an ATI program site where recruitment is to occur, with instructions to provide a copy of the letter to all probation, parole or ATI program officers at each such site. The researchers have taken steps to assure privacy and the confidentiality of the information provided by the participants. These steps include obtaining a Certificate of Confidentiality and the assurance of Fortune Society that it will not disclose any information to others or seek access to it. Further, probation, parole or ATI program sites will not have any documents linking the participants name to any information given to the researchers. In the criminal justice system, however, there are no absolute guarantees of confidentiality. If participants have signed a waiver of confidentiality or release (or sign one in the future), the holder of such a waiver or release may seek to obtain information from the researchers. The researchers will resist disclosure of such information by all lawful means.

All study staff will undergo a common training that includes the impetus, goals, organization, and rules/regulations under which the study operates; this common "base" training also covers: (1) ethical issues in research; (2) establishing and maintaining rapport with participants; (3) obtaining informed consent; (4) addressing participants' concerns with confidentiality and handling sensitive situations; (5) obtaining accurate tracking information; (6) managing distress and conducting crisis and enhanced referrals; and (7) detecting, handling, and reporting adverse events. This common, general training is supplemented with more specialized topics and scenarios during trainings dedicated to specific roles/tasks (e.g., Fortune providers or RAs who deliver HIV/STI results) as appropriate.

Confidentiality of in-depth interviews with Probation, Parole or ATI program staff and Fortune providers: In addition, for the purposes of improving this program, participants will be audio taped in the sessions and/or audio taped during an interview session. Only research staff will review these tapes. The tapes are for research and training purposes only, and will be erased after the study is completed. Interviewing records will only be shared with the research team. Recordings will be downloaded to a secure and password protected for storage and then erased from the digital recorder. The recording will be reviewed by a research assistant for accuracy and thematic analysis. Any identifying from the defined by the Health Insurance and Portability Act (HIPAA) will be immediately deleted from the recording. All digital recordings will be destroyed at the end of the study after they have been transcribed and the transcriptions have been checked for accuracy. All electronic files transcripts and thematic analyses of transcripts will be encrypted and password protected.

Disclosure Protocol: By virtue of the study population, it is anticipated that some of the subjects may disclose past or current engagement in law breaking activity (e.g., sex trading, selling drugs, theft, etc). Following protocols used in the pilot study and prior SIG research with drug-involved offenders, research staff and Fortune providers will inform participants of the limits of confidentiality at the start of session. Research staff will not release any information about participant to anyone unless there is a possibility of "imminent harm to self or others" and/or, through group or individual discussions it becomes clear that a child is in danger or is experiencing physical abuse or neglect. Dr. Gilbert has secured a Certificate of Confidentiality from DHHS to avoid subpoena regarding confidential information from subjects upon receipt of funding for this study.

Suicidal and Homicidal Respondents: Confidentiality cannot be maintained if a participant is homicidal or demonstrates intent on seriously injuring another person. Similarly, a respondent may indicate that he is considering hurting himself. Following protocols used in prior SIG studies intervention trials with populations at elevated risk for HIV/STI transmission, we will inform the appropriate agency (e.g., law enforcement, hospital) in these cases. Research assistants will be trained to handle either situation in the event it occurs, and these procedures will be described in the informed consent forms and reviewed with all participants during consent procedures. Like the procedures for referrals, data regarding implementation of procedures to address suicidal/homicidal participants will be collected and analyzed in a parallel manner to investigate the effects of the research process itself on utilization/involvement with the service system and other outcomes.

Child Abuse/Neglect: By law, the privilege of confidentiality does not extend to information about the sexual or physical abuse or neglect of a child. If a participant makes statements from his personal knowledge, which if correct, would render a child abused or neglected, New York City's Administration for Children's Services (ACS) will be notified. The PI will make every effort to attempt to ensure the safety of participants. Participants will be informed of these mandates during administration of the informed consent protocol. Like the procedures for referrals and suicidal/homicidal participants, data regarding implementation of procedures to address child abuse/neglect will be collected and analyzed in a parallel manner to investigate the effects of the research process itself on utilization/involvement with the service system and other outcomes.

11. Potential Risks

Screening, assessments, and interventions should pose minimal risks to subjects participating in the study. Subjects may become embarrassed or uncomfortable while being asked questions and there is some risk that discussing sensitive topics will cause distress. Because subjects will take part in a group intervention, there is a risk that other group members may divulge confidential information to people outside the group. We will address this by asking everyone to respect the confidentiality of the other participants. Participants will be advised that conversations about HIV and safer sex negotiation may anger their male partners. This anger may put them at risk for emotional, physical and/or sexual abuse or abandonment of the relationship. Participants will be advised to observe any signs of such anger in their partners and to discuss them with study staff immediately.

Study staff will be trained to provide crisis intervention and referral for such situations as follows: All project staff will be trained to observe verbal and non-verbal signs that may indicate that the respondent is emotionally distressed. In cases where the respondent is exhibiting distress, the data collector, recruiter, or facilitator will acknowledge that the questionnaire or group session can raise troubling issues for respondents and ask the respondent how she is feeling and whether or not she wants to take a break from the interview/group. If the respondent continues to exhibit distress, the data collector/recruiter/facilitators will ask her if she wants to terminate the interview or session and encourage her to discuss the issues further with any existing counselor. If a respondent seems unresponsive or unwilling to go to the counselor, or if there is no current counselor, the researcher staff will offer her a range of counseling options in the local community, using a resource manual developed for this project that contains local services and resources with upto-date contacts who have indicated a willingness to serve women from our

study population. Project staff will refer participants who request such services to professionals who we have identified in the community. After assisting the participant in exploring the counseling, residential and/or legal services available, the facilitator will offer to assist the participant in locating appropriate services and will provide enhanced referrals (e.g., calling the organization and assisting in making an appointment). If the participant is undecided about needing help and is not ready to take a referral, the facilitator will give the participant a business card with the phone number of the Project Director and encourage her to call if she would like a referral in the future. Acute emergency distress referrals will be directed to the designated counselor or social worker, and the Project Director and Dr. Gilbert, who is a certified social worker, will be alerted. In the event that a respondent indicates to research staff that she is suicidal or homicidal, a designated clinic counselor or a SIG certified mental health professional will be informed and available for crisis intervention. At the end of all assessment and intervention sessions, whether or not complete, staff will ask anxious or troubled participants for permission to discuss their discomfort with their treatment program counselors. If permission is granted, research staff will walk distressed participants directly to the counselor. Any participant choosing not to be referred to their counselor will receive counseling from trained certified professional mental health counselors on the research team, including the PI, Co-Investigators, and the Project Director, available as back-up to treat and make enhanced referrals for distressed participants.

Additionally, risks from this project may arise from obtaining specimens, or from conducting a rapid oral HIV test. Women may feel uncomfortable providing specimens for STI testing, providing an oral swab to test for HIV, or receiving pre- and post-test counseling for HIV. This project also involves risks that are associated with positive STI and HIV testing. The possible diagnosis of an STI or HIV and partner notification could cause emotional distress. Although unlikely, breach of confidentiality regarding STI status, HIV serostatus, sexual behaviors and relationships resulting from participation in this intervention may cause participants some level of psychological, social or financial distress. There is a possibility of unauthorized disclosure of sensitive information shared during screening, intervention sessions, or assessments. To minimize the likelihood of unintended disclosure and to maintain confidentiality safeguards, research staff will be trained to remind participants that they may at any point request their data be destroyed without question. Additionally, the NYC Departments of Probation, Parole and designated ATI program sites have indicated in letter of supports that they will not request any participant information obtained through the study. By virtue of the study population, it is anticipated that some of the subjects may disclose past or current illegal activity (e.g. sex trading, drug activity, theft, etc.). Other possible types of information disclosed are intimate partner violence, suicidal or homicidal ideation, or
child abuse/neglect. Following protocols used in prior SIG research with drug-involved offenders, research staff will not release any information about participants to anyone unless there is a possibility of imminent harm to self or others and/or actual or suspected child abuse or neglect.

11.1 Vulnerable Populations

The proposed study will not involve participants who meet the criteria for classification as prisoners. As the study will involve participants who are in probation, parole or ATI programs, there is a likelihood that some participants may become incarcerated. If a participant becomes

incarcerated or detained, the adverse events protocol described below will be followed, and the participant's involvement in the study will be discontinued.

12. Adverse events, Protocol Violations and Procedural Variations

A policies and procedures manual for the project will specify immediate data monitoring and response for anticipated health or mental health issues. Timely data analysis will ascertain if addressable prevalent conditions occur. Data on adverse events and psychological distress will be systematically collected during the trial and reported to the PIs and the Columbia University IRB in order to secure appropriate management of the adverse events as well as to have full documentation of the events for all conditions. The following steps detailed below will be used. All research staff will have completed the Columbia University Office of Projects and Grants' Human Subjects training course, which has received approval from DHHS Office of Human Research Protections to supersede the NIH training course on Protection of Human Research Subjects. The Columbia University training also includes compliance with the Health Insurance Portability and Accountability Act of 1996 (HIPAA). As part of SIG's standard operating protocols, all SIG field, quality assurance, and management staff must complete an intensive, day-long, structured training program on detecting, addressing, and reporting adverse events before they are allowed to participate in any research. This training which will be conducted by the PIs will also cover how to handle challenging situations, including how to respond to distressed participants and participants who are experiencing life threatening IPV. Also, each research study at SIG conducts an annual 3-hour meeting, first reviewing human subjects protection principles and study-specific procedures, followed by discussion of adverse events for the study over the past year, and role-play exercises which provide a booster to enable staff to handle future adverse events knowledgeably, appropriately, and expeditiously.

Serious adverse events include death, a life-threatening event, an event resulting in hospitalization, prolongation of hospitalization, disability, and incarceration. Serious adverse events, regardless of whether or not they are study related, will be reported to the Columbia University IRB. Other adverse events include breaches of confidentiality (both intentional - e.g., mandated reporting of suicidal/homicidal participants - and unintentional), and non-life threatening psychological distress. The PIs will closely monitor adverse events. The investigative team is aware that the study sample is likely to experience a relatively high incidence of adverse events due to their past/current history of law-breaking behavior, rates of recidivism/re-incarceration reported in the literature, and the high levels of service needs noted earlier in this application. Drawing upon past and ongoing SIG studies, the investigative team designed two forms, (1) Adverse Event: Initial Report and (2) Adverse Event: Full Report, to document adverse events, staff responses, and disposition. The procedures used to track, report, and examine adverse events are described below.

Protocol to Handle Each Adverse Event: Study staff and Fortune providers will identify, manage and document all adverse events. These events may be identified by RAs, the Project Director, PI, or other project staff, including quality assurance staff reviewing audio recordings of interviews, or they may be reported by participants. The following steps will be used to monitor, track, and document procedures used to address each adverse event and subsequent disposition. Within 12 hours of an adverse event being reported/detected, an Adverse Event: Initial Report study report form will be completed by the study staff member or Fortune provider who identified the event. The report form will include date, description of the event, duration,

severity, measures taken to ameliorate the adverse event, and disposition-related information (e.g., time spent providing referrals and type of referral (domestic violence shelter, mental health or substance abuse treatment, etc.). The report form will be reviewed and signed by the immediate supervisor who will be responsible for ensuring that appropriate actions have been taken. Within 24 hours of the adverse event, the research staff member or provider who identified the adverse event will discuss the event and response with the Project Director (or the PIs if the Project Director is unavailable) in order to ensure adherence to the protocol for handling the event. (3) Within 48 hours of the adverse event, the investigative team, the Project Director, and any involved staff will ascertain whether the event was related to participation in the study.

An Adverse Event Full Report form will be completed by Drs. Gilbert and El-Bassel. This full report will contain an attached copy of the initial adverse event form, a summary of information obtained from other documentation and sources that clarify the nature of the event and outcomes, and the determination of whether the event was study related and by what criteria such determination was made. (4) Drs. Gilbert and El-Bassel will be responsible for reviewing the event occurrence with the appropriate staff to ensure that an adequate response is provided to the participant. Any serious adverse event whether or not related to the study will be reported to the Columbia University, and the NIDA project officer for the study. The initial adverse event report will be submitted to Columbia University's IRB. This will be followed by submission of the full report to the IRB. In the event that a participant withdraws from the study or the investigator decides to discontinue a participant due to an adverse event, the participant will be monitored by the investigators via ongoing status assessment until (a) a resolution is reached, (i.e. a problem requiring hospitalization is stabilized with no further change expected, or (b) the event is determined to be unrelated to the study.

Oversight and Review of Adverse Events: Every two months, the entire investigative team will review all adverse event data to date to determine if systematic trends exist among adverse event data to warrant changes to study protocols and procedures. Any proposed changes will be reviewed with staff at the study sites, and, if needed, external consultant (e. g., other senior investigators conducting federally-funded research on drug abuse and/or criminal justice). The investigative team will also conduct systematic analyses of adverse event data (e.g., associations among frequency, type, severity of adverse events; participant characteristics; and operational aspects of research such as time point, gender of interviewer) to inform future studies with participants in probation, parole or ATI programs as well as recognizing such activities represent a potentially under- researched area of inquiry and scientific endeavor unto itself.

Protocol Violations and Procedural Variations – protocol violations and procedural variations from established study protocols and procedures will also be documented by the Project Director and the PI and reported to the IRB using the Protocol Violation and Procedural Variation Forms. A binder of procedural variations will also be maintained by the Project Director.

13. Data Safety and Monitoring

The Data Safety and Monitoring Board (DSMB) will oversee and provide advice to the PIs on the continued scientific integrity and safety of the implementation of the trial. At each meeting the board will review the status of recruitment, retention, successes, barriers and challenges of the implementation of the trial, adverse events and the reporting procedures of adverse events.

Two interim analyses will be conducted in addition to the planned “final” analyses at the conclusion of the proposed study. These interim analyses will be conducted when 33.3% and 66.7% of the planned sample size have passed their 12-month follow-up assessment time point window. The DSMB will review findings from the interim analysis and determine whether the study needs to be terminated for safety reasons. Data will be sent to the DSMB a week prior to the meeting. The Board will meet twice a year. The meetings will be face-to-face. Minutes taken at the meeting will be archived. We have included a statement in the DSMP that the proposed Board members have no Conflict of Interest with the study. The DSMB will consist of four expert members in the field who are experienced with HIV treatment, substance abuse treatment, and vulnerable populations. The DSMB will meet twice a year. The DSMB will consist of three expert members in the field who are experienced with HIV treatment, substance abuse treatment, and vulnerable populations. Specifically the members will include:

1. Wendee Wechsberg, PhD, is the Senior Director of the Substance Abuse Treatment Evaluations and Interventions Research Program at RTI International. Since its inception in 1999, this program has evolved to include a multimillion dollar yearly portfolio. She is also Adjunct Professor at the School of Public Health at UNC, Chapel Hill, and Adjunct Associate Professor of Psychiatry at Duke University School of Medicine. She has conducted multiple NIH-funded clinical trials testing gender specific HIV interventions with different populations of women drug users.
2. Sandro Galea, MD, is a Professor of Epidemiology and Public Health at Columbia University School of Public Health. He is an expert in addiction medicine and HIV care among drug users. Scarlett Bellamy, PhD is an Associate Professor of Biostatistics at University of Pennsylvania. She is an expert in developing data analytic plans and analyzing outcome data for clinical trials and will perform the interim analyses.
3. Jack DeHovitz, MD is a Professor of Medicine at SUNY Downstate Medical Center. He has extensive experience conducting clinical research with HIV-infected women drug users.

Interim Analyses and Stopping Rules: Two interim analyses will be conducted in addition to the planned final analyses at the conclusion of the proposed study. These interim analyses will be conducted when 33.3% and 66.7% of the planned sample size (N=140 and N=280 women respectively) have passed their 12-month follow-up assessment time point window. Several considerations and/or constraints were balanced in deciding upon key aspects of interim analyses. We restricted the number of interim analyses to 2 in order to prevent too great a threat to valid statistical inference due to a theoretical need to adjust analyses to avoid Type I error inflation from multiple comparisons using the same data. Interim analyses will be conducted at the selected information rates (i.e., the 33.3% and 66.7% values) based on 12-month follow-up duration period as per recommendations in Methodological Challenges in Biomedical HIV Prevention Trials published in 2008 by the Institute of Medicine (IOM). Specifically, in the chapter entitled Interim Monitoring and Analysis of Results, the IOM recommended that stopping HIV prevention trials based on positive interim results should require evidence of a sustained impact. Thus, interim analyses will utilize complete data over the longest follow-up duration, i.e., the 12-month assessment time point. The Principal Investigators and Co-Investigators will be unblinded to the research, whereas the study staff will be blinded to the interim analysis. The interim analysis will be conducted by the statistician on the study and the results will be reported to the PIs. With respect to efficacy, stopping rules following an interim analysis focus on demonstrable benefit among the sample and data accrued to date.

At each interim time point as well as after study completion, analyses will follow the data analysis plans for primary aims described in the original proposal. Interim analyses will focus on the study's primary aims. If at any of the interim analyses the statistical significance for all biological outcomes related to primary aims (i.e., cumulative STI incidence), exceeds the a prior a < .05 criterion at that time point, the study will be stopped since the inference will be that significant benefit of assignment to one study arm over the other will have already been demonstrated. We note that a represents two-sided hypothesis testing since demonstrating benefit with respect to disease prevention of one intervention over the other (regardless of the content of that intervention) is of public health value and importance. With respect to futility, stopping rules are concerned with effect size estimates indicating a null result is inevitable given the planned total sample size. Stopping a study in such circumstances avoids additional expenditure of time, fiscal, and human resources that would nevertheless ultimately result in lack of evidence of superiority of one intervention over the other. At each interim analysis time point, a post hoc power analyses will be conducted to determine the minimum sample size needed to achieve 80% power to reject the null hypothesis given point estimates and standard errors of efficacy estimates using data collected to date. If the estimated minimum sample size for power analyses using a = .05 exceeds the planned enrollment of N=420 women for all of the biological and self-reported behavioral outcomes associated with the primary aims, the investigative team will notify the NIDA Program Official (PO) of such findings. Based on the magnitude of the difference between the estimated minimum sample sizes needed for 80% power and targeted sample size, in addition to recruitment rates to date, the investigative time and NIDA PO will discuss and agree whether the study should be stopped due to reasons of futility or whether modifications to the study design/protocols (e.g., increasing planned enrollment, hiring of additional recruitment staff, etc.) can be implemented to avoid futility.

14. Potential Benefits

Potential benefits to study participants are learning skills and information that may lead to decreased risk of contracting and transmitting HIV and other STIs. The benefit to society is that the investigators may develop a better understanding of which interventions are most useful for promoting safer sex among justice-involved women.

15. Alternatives

N/A

16. Research at External Sites

The investigative team has conducted several randomized controlled trials (RCTs and prevention and services research in criminal justice systems, including three recent or current RCTS with the NYC Department of Probation. They have established strong collaboration with the NYC New York City Department of Probation Sites: Participants will be recruited from probation sites throughout NYC. We will also recruit participants from parole and designated ATI program sites. We will particularly target sites that serve low income minority communities in NYC most heavily affected by HIV/STIs. The investigative team has collaborated with Probation on three HIV research projects since 2008. Projected Enrollment: In 2012 there were at total of 3,115 Black women at the 5 DOP sites. Given the annual pool of Black women on probation from the five study sites (N=3,115) as of January 2012 and the given estimated number of 1,250-

1500 additional Black women per year who will be placed on probation during the enrollment period at the 5 sites (N=3,993), we project a total recruitment pool of 7,045 Black women on probation specifically who may be approached for screening. Of this pool, we estimate 70% (N=5,607) will agree to complete a screening interview based on our current and recently completed studies in probation of whom 35% (1,726) will meet eligibility criteria based on studies summarized earlier and enroll in the study. This number will augmented by women on parole or an ATI program. This flow of potential participants will be ample to achieve a sample size of 420 women.

Staff from Fortune Society, which is the largest probation contract provider in NYC, and also provides services to individuals on parole and other ATI programs, will be delivering both interventions for this study (see letter of support) either at their site or at one of the probation, parole or ATI program sites. Fortune Society does not have an IRB or access to an IRB. Stanley Richards, our administrative contact at Fortune Society, has indicated that they will accept Columbia as the Sole IRB for review and approval of this study. The NYC Departments of Probation and Parole, and leadership personnel from designated ATI program locations, will provide space for recruiting participants and conducting assessments at probation, parole and ATI program sites located throughout in New York City, however, they will not have any contact with human subjects for this study. New York City (NYC) Department of Probation, Parole and ATI Sites: In New York State, probation, parole or ATI are court-ordered sentences to community supervision with certain conditions. NYC operates probation and parole departments, and ATI programs, with funding and oversight by the New York State Division of Probation and Correctional Administration (DPCA). Each of the five NYC boroughs has probation, parole and ATI program reporting sites, where offenders under supervision report to probation, parole and ATI program officers with frequencies ranging from weekly to several times a year.

Social Intervention Group- Community Research Site: The Social Intervention Group (SIG) at the Columbia University School of Social Work (CUSSW) has a new community space where we conduct all assessments and intervention sessions, which is located within a 10-minute walk of our administrative offices at CUSSW. This community space was outfitted for this study and contains two gender specific assessment rooms that are equipped to conduct the Audio Computerized Assisted Structured Interviews (ACASI), a bio-specimen lab, which is equipped to process, store and ship specimens; four private offices where the face-to-face interviews and pretest and post-test HIV/STI counseling are conducted; four intervention rooms – one of which is outfitted with PCs to conduct Web based multimedia interventions. These intervention rooms can comfortably fit up to 14 people and are equipped with the audiovisual equipment, flip charts and all other materials specific to each intervention. This community space also has two office rooms with workstations to accommodate project staff while they are in between sessions and locked file cabinets to store confidential materials.

17. Columbia as Lead Institution

Columbia University is the lead and sole institution implementing this project. Letters of support from Fortun[illegible] Society and the New York City Departments of Probation and Parole, and designated ATI program locations are attached. As Fortune Society does not have an IRB, we applied for and received IRB coverage through Columbia University. We have also secured an IIA Agreement (FWA00022811).
