# Supplementary material for: Effectiveness of a Culturally Tailored HIV and Sexually Transmitted Infection Prevention Intervention for Black Women in Community Supervision Programs: A Randomized Clinical Trial
Source: JAMA Netw Open. 2021 Apr 9;4(4):e215226. doi: 10.1001/jamanetworkopen.2021.5226 (PMC8035652; doi:10.1001/jamanetworkopen.2021.5226)
Supplement: Supplement 2. — Data Sharing Statement [file jamanetwopen-e215226-s002.pdf]

Data Sharing Statement

Gilbert. Effectiveness of a Culturally Tailored HIV and Sexually Transmitted Infection Prevention Intervention for Black Women in Community Supervision Programs. *JAMA Netw Open*. Published April 09, 2021. doi:10.1001/jamanetworkopen.2021.5226

Data
Data available: Yes
Data types: Deidentified participant data, Data dictionary
How to access data: Please contact Louisa Gilbert at lg123@columbia.edu
When available: beginning date: 01-01-2022

Supporting Documents
Document types: Statistical/analytic code, Informed consent form
How to access documents: Please contact Louisa Gilbert at lg123@columbia.edu
When available: beginning date: 01-01-2022

Additional Information
Who can access the data: Please contact Louisa Gilbert at lg123@columbia.edu
Types of analyses: Data will be made for a specified purpose
Mechanisms of data availability: Data will be made after approval of a proposal by the Principal investigators or a designated representative.
